# Cost-utility analysis of prenatal diagnosis of congenital cardiac diseases using deep learning

**DOI:** 10.1186/s12962-024-00550-3

**Published:** 2024-05-22

**Authors:** Gary M. Ginsberg, Lior Drukker, Uri Pollak, Mayer Brezis

**Affiliations:** 1 Braun School of Public Health, Hebrew University, Jerusalem, Israel; 2 HECON, Health Economics Consultancy, Jerusalem, Israel; 3 Department of Obstetrics and Gynecology, Rabin-Belinson Medical Center, Petah Tikva, Israel; 4 School of Medicine, Faculty of Medical and Health Sciences, Tel-Aviv University, Tel Aviv-Yafo, Israel; 5 Pediatric Critical Care Sector, Hadassah University Medical Center, Jerusalem, Israel; 6 Faculty of Medicine, Hebrew University Medical Center, Jerusalem, Israel; 7 Center for Quality and Safety, Hadassah University Medical Center, Jerusalem, Israel

**Keywords:** Prenatal screening, Ultrasound, Congenital cardiac disease, Deep learning, Cost-utility analysis

## Abstract

**Background:**

Deep learning (DL) is a new technology that can assist prenatal ultrasound (US) in the detection of congenital heart disease (CHD) at the prenatal stage. Hence, an economic-epidemiologic evaluation (aka Cost-Utility Analysis) is required to assist policymakers in deciding whether to adopt the new technology.

**Methods:**

The incremental cost-utility ratios (CUR), of adding DL assisted ultrasound (DL-US) to the current provision of US plus pulse oximetry (POX), was calculated by building a spreadsheet model that integrated demographic, economic epidemiological, health service utilization, screening performance, survival and lifetime quality of life data based on the standard formula:

$${\rm CUR} = \frac{{\text{Increase in Intervention Costs}} - {\text{Decrease in Treatment costs}}}{{\text{Averted QALY losses of adding DL to US}} \ \& \ {\rm POX}}$$

US screening data were based on real-world operational routine reports (as opposed to research studies). The DL screening cost of 145 USD was based on Israeli US costs plus 20.54 USD for reading and recording screens.

**Results:**

The addition of DL assisted US, which is associated with increased sensitivity (95% vs 58.1%), resulted in far fewer undiagnosed infants (16 vs 102 [or 2.9% vs 15.4%] of the 560 and 659 births, respectively). Adoption of DL-US will add 1,204 QALYs. with increased screening costs 22.5 million USD largely offset by decreased treatment costs (20.4 million USD). Therefore, the new DL-US technology is considered “very cost-effective”, costing only 1,720 USD per QALY. For most performance combinations (sensitivity > 80%, specificity > 90%), the adoption of DL-US is either cost effective or very cost effective. For specificities greater than 98% (with sensitivities above 94%), DL-US (& POX) is said to “dominate” US (& POX) by providing more QALYs at a lower cost.

**Conclusion:**

Our exploratory CUA calculations indicate the feasibility of DL-US as being at least cost-effective.

**Supplementary Information:**

The online version contains supplementary material available at 10.1186/s12962-024-00550-3.

## Introduction

Congenital heart diseases (CHD) are the most common type of congenital defect, accounting for nearly one-third of all major congenital anomalies [1, 2]. CHD, and most notably, critical CHD (cCHD), is the leading cause of mortality and morbidity from birth abnormalities worldwide [3, 4], accounting for more than 200,000 deaths annually [3]. In developed countries, more than half of the total cost attributed to all birth defects combined is currently associated with care of CHD [5].

CHD is considered major if it requires cardiac surgery or catheter intervention or results in death in the first year of life. It is defined as critical if these occur in the first 28 days of life [6]. Critical CHD conditions include valvular atresia or severe stenosis, coarctation of the aorta, transposition of the great arteries, total anomalous pulmonary venous connection [4] and many others. Within this group, the outcome varies considerably, from a guarded outlook, such as in hypoplastic left heart syndrome (HLHS) or interruption of the aortic arch, to conditions with better outcomes, such as complete transposition of the great arteries (TGA) [6].

Primary prevention of CHD is possible to some extent via improved diabetic control, switching to nonteratogenic medicine for treating epilepsy and possibly iron and folic acid supplementation. Unfortunately, less than one half of CHD (especially minor CHD) are detected prenatally [7–19], although detection rates vary depending on the type of defect [17, 19–21], the examiner skill [22], and specific population [23, 24].

Increasing detection via prenatal diagnosis of CHD (and subsequent possible timely treatment), should result in a lower morbidity and mortality [25–31], only partly due to possible elective terminations of pregnancy, Prenatal diagnosis allows for family preparation, facilitates counselling, shared decision-making, planning for optimal neonatal intervention and medical care after delivery [31, 32], including the transfer of deliveries to a tertiary care center with resources to manage critically ill newborns [14, 33, 34], resulting in fewer and less severe accompanying neurodevelopmental disabilities [20, 31] and improved childhood developmental milestones.

Almost 30% of newborns affected with CHD are diagnosed late [35] and are more likely to experience hemodynamic compromise, resulting in prolonged hypoxemia to vital organs. The resultant untimely medical-surgical intervention results in elevated morbidity and mortality rates, including irreversible pulmonary hypertension [36, 37].

A study of a pediatric population with pulmonary hypertension reported high readmission rates and use of expensive intensive care unit resources [38]. Overall children with CHD incur 23% of total hospitalization costs globally, while accounting for only 4.4% of all hospital admissions [39]. Importantly, the distance between the place of birth and a cardiac center has been shown to be correlated with neonatal death rates [40]. Clearly, delayed diagnosis of CHD imposes a large cost burden on health services.

We did not include universal fetal echocardiographic (UFE) screening in our analysis for the following reasons:-

- Use of UFE would impose a huge and prohibitive workload that is absolutely impossible to handle within the constraint of the current workforce of echocardiographers.
- According to a recent paper [41], even in the case of pregnancy diabetes, UFE was shown to be cost effective only when first-trimester Hb A1c levels were above 9.0% but not when normal (> 6.5%). Universal fetal echocardiograms became both cost saving and more effective only when the probability of congenital heart disease reached 14.48% (15.4 times the baseline risk!).
- In any case, the major focus of paper was the effects of adding Deep Learning to the prenatal and postnatal diagnosis protocols that are currently in effect.

Besides manpower constraints, adding universal echocardiographic screening of newborns (to routine prenatal screening) is also unlikely to be cost effective. This is due not only to the high screening costs associated with echocardiography but also to the diminished pool (because of initial prenatal screening) of as-yet undetected cardiac abnormalities. Adding low-cost universal pulse oximetry (POX) screening to newborns is more likely to be cost effective. A UK modelling study [4] reported an incremental cost of approximately 41,000 USD (at 2009 price levels) per timely diagnosis of POX and a routine clinical examination in a population in which antenatal screening for CHDs already existed.

Routine implementation of POX was expected to be cost-effective in many studies [4, 42–47], including a Dutch study where homebirths were predominant [48]. However, resultant potential treatment cost savings and quality of life improvements, which would have resulted in a full cost utility analysis, were rarely included in such studies. Likewise, many previous cost-effective ultrasound (US) studies were limited to reporting either the cost per detected CHD case [48–50] and/or to the diagnosis of a specific ailment, such as coarctation of the aorta [51]. The cost per detected case was as high as $113,000 USD (at 2012 price levels) in the USA [49], with an antenatal ultrasound that includes five cardiac axial screening views having the lowest cost per detected case [52, 53].

For our study purposes, we defined severe congenital heart disease (sCHD) as a diagnosis of either critical or major CHD. For the sake of completeness in measuring all the potential benefits, we also included screening effects on minor CHD (mCHD), which include ventricular septal defects, atrial septal defects and bicuspid aortic valves. mCHD are more challenging to diagnose prenatally, in addition to prenatal diagnosis possibly having little impact on morbidity and neonatal mortality.

Recently, artificial intelligence-driven deep learning (DL) has been explored as a complement to and as an enhancement of routine US (referred to as DL-US) through its ability to increase the sensitivity of prenatal discovery of CHD [54, 55]. As a guide for policy-makers in deciding whether to adopt the new technology (DL-US), this study aimed to carry out full cost-utility analyses (CUAs) of various combinations of US (see Additional file 1: Appendix Ia for a fuller description), POX (Additional file 1: Appendix Ib) and artificial intelligence-driven DL-US [see Additional file 1: Appendix Ic] by modelling the many diagnoses-specific survival gains, quality of life gains and treatment costs.

## Methods

The Cost-Utility Ratio (CUR) was based on applying the interventions to the Israeli national population on a national level using a lifetime and societal perspective. CURs (compared to the “null” of no screening) of various combinations of US, POX and DL-US, were modelled and calculated using an Excel based spreadsheet that integrated demographic [56–58], economic [59–64], epidemiologic [65–68], screening efficacy [42], health service utilization [72], survival [58, 59] and quality of life [52, 59, 73–76] data. Full details of how survival gains were modelled are shown in Additional file 1: Appendix II, with the full complex (essentially Markov) methodology of calculating inputs into the cost-utility model, being described in Additional file 1: Appendix III.

Firstly, we compared each possible combinations of interventions (eg: US, DL-US, POX etc.) with the “null” (do-nothing scenario). The “null scenario” despite having no intervention costs does invoke treatment costs and Quality Adjusted Life Year (QALY) losses due to morbidity and mortality. Using the following formula, based on the principals of generalized cost-effectiveness analysis [77] we calculated the Average Cost Effectiveness Ratios (ACER): where

$${\rm ACER} = \frac{{\text{}}- ({\text{Treatment costs with ``null''}} - {\text{Treatment Costs with intervention}})}{({\text{QALY losses under ``null''}} - {\text{QALY losses due to the intervention}})}$$

Next a similar formula was used to calculate the Incremental Cost Effectiveness Ratios (ICER) of comparing two interventions A and B (eg: adding DL to the current practice of US + POX). Where

$${\text{ICER}}\,{\text{ = }}\,\frac{{{\text{Increase in Intervention Costs}}^*- \left( {{\text{Decrease in Treatment costs}}^{**}} \right)}}{{{\text{Averted QALY losses of substituting intervention A for intervention}}\,``{\text{B}}"^{***}}}$$

*Cost of Intervention A—Cost of Intervention B.

**Treatment cost under intervention B—treatment cost under intervention A.

***QALY losses under intervention B—QALY losses under intervention A.

All costs are in USD (at 2022 price levels), based on the average exchange rate of 3.36 NIS to USD [78]. Future costs and utilities were discounted using a rate of 3% per annum. In the absence of Israeli specific guidelines, interventions were deemed [79] to be cost saving, very cost effective, cost effective, or not cost effective when treatment savings exceeded intervention costs, CUR < GDP (Gross Domestic Product) per capita, GDP per capita < CUR < 3 × GDP per capita and CUR > 3 × GDP per capita, based on Israel’s GDP per capita in 2022 of approximately 54,800 USD [80]. Where the CUA could either be the average (when comparisons are with the null-ACER) or incremental (when comparisons are between interventions-ICER) CUR.

Calculating the numerous CURs required the following data to be collected:-

- Initial number of pregnancies, losses at end of first trimester, miscarriages, elective terminations, stillbirths, livebirths and mulytiple births.
- Screening performance, sensitivities, specificities and costs of various combintions of interventions.
- Survival data and lifetime treatment (or abortion) costs by gender, CHD diagnosis at time of diagnosis (prenatal, less than or more than 24 h).
- Healthy Adjusted Life Expectancy (HALE) by gender, CHD diagnosis and time of diagnosis.

We assumed a “baseline” based on the few reported US studies [69–71] that were carried out in routine settings in busy primary care units. These were characterized by lower standard operators working and devoting less than adequate time to the US. These will be subsequently referred to as “routine reports”, which contrast with higher standard US reports carried out under “research study” conditions that are characterized by prospective supervised academic research in referral centers.

In this model, the baseline DL-US sensitivity and specificity were assumed to be 95% and 96%, respectively. The baseline definition enabled the exploration of the CUR of all the potential strategies, viz: null (i.e., doing nothing), US, POX at birth, US plus POX, DL-US, and DL-US plus POX.

Our major focus was evaluating a possible future operational change where the current operational screening of US plus POX would be replaced in the future by DL-US plus POX. Since there is a dearth of studies reporting operational data for DL-US, we ran the model over a wide range of expected DL-US sensitivities (from 80 to 99%) and expected specificities (from 90 to 100%) for the following three scenarios:

A. Routine: Based on data from the few “routine reports” of US studies that were based on actual real operational data. A cost per DL-US screen of $144.82 was based on Israeli US costs of $124.42 plus $20.40 for reading and recording the screen.
B. Routine High Cost: Based on data from the few “routine reports” of US studies. The cost per DL-US screen of $248.84 was assumed to be double that of US screens to reflect the pricing of the new technology to cover development costs.
C. Routine High Costs & High Performance: Based on data from the numerous US performed under “research study” conditions, that reported better operational data (i.e. higher sensitivities) than did those based on the few real-life “routine reports” of retrospective studies. To achieve these higher operational standards, we assumed that double the amount of time would be allocated for the US screen (costing $248.84) plus an additional 25% of the original time for extra supervision ($31.11), for a total screen cost of $279.95. A cost per DL-US screen of $300.43 was based on the $279.95 US cost plus $20.48 for reading and recording the screen.

One way sensitivity analyses of numerous variables on the baseline CUR were carried out so as to identify variables that effect the CUR the most. Ranges of the two variables with the highest elasticities with respect to the CUR, were included in a 2 by 2 matrix of CUR outcomes.

## Results

### Demographics

Based on a backwards calculation from birth data, in 2022, there were an estimated 199,935 pregnancies, with an early pregnancy loss of 12% [57] resulting in 175,943 viable pregnancies by the end of the first trimester, when the nuchal translucency scan is offered and taken up by nearly all women in Israel. There were an additional 3151 elective terminations of pregnancies [56, 80, 81], 3% [57] foetal losses after the US and 0.345% stillbirths [82], resulting in 167,031 birth episodes and 181,269 new-borns [58].

### Survival in CHD patients

A sample of just over half of all sCHDs in Israel was used (Additional file 1: Appendix IV). Weighted survival rates based on prenatal diagnosis were non-significantly greater than those based on postnatal diagnosis (88.3% vs 87.1%; not sig). However, a survival advantage was found in favor of prenatal (vs. postnatal) diagnosis for several but not all CHDs: Left heart obstruction (93.3% vs 80.9%; not sig), HLHS (71.1% vs 61.8%; p < 0.001) and TGA (96.2% vs 92.0%; p < 0.001). Conversely, for truncus arteriosus survival, there was a paradoxically lower survival rate for prenatal diagnosis (57.9% vs 91.5%; p < 0.0001),—possibly because more severe conditions may be more easily detected in utero. The postnatal survival rate was split into 87.2% and 87.0% for diagnoses ≤ 24 h or > 24 h, respectively.

For mCHD patients, the one-year survival rates were 93.40%, 96.44% and 96.40% for prenatal, postnatal <  = 24 h and postnatal > 24 h, respectively.

### Treatment costs

For the first year of life, treatment costs were $13,657 and $8,232 for sCHD and mCHD, respectively. The lifetime discount costs for sCHD patients diagnosed prenatally, < 24 h and >  = 24 h were $220,570, $214,249 and $213,259, respectively, for males but were greater for females, $242,294, $236,014 and $235,551, respectively (due to increased life expectancy).

For mCHD, the discounted lifetime treatment costs were $163,672, $186,079, and $185,646 for males and $176,843, $198,636 and $198,216 for females diagnosed prenatally, < 24 h and >  = 24 h, respectively.

### Screening performance

Three “routine reports” (from 2015 to 23) for sCHD reported [69–71] sensitivities ranging from 33.3% to 79.3% (weighted average 58.1%), alongside reported specificities of 100%. This performance was far lower than the 79.9% sensitivity, and a similar 99.95% specificity that were found in many publications [82] based on the use of US and carried out under “research study” conditions (see Additional file 1: Appendix V).

For mCHD (from 2015 to 2023), we excluded the two lone sensitivities of mCHD from “routine reports” due to lack of homogeneity (reporting 50% and 2.7% sensitivities). Instead, our model estimated a sensitivity for mCHD of 23.0%, based on the relative magnitudes of sensitivity for sCHD reported under “research study” (58.1%) and “routine reports” (79.9%) conditions multiplied by 31.6%. being the sensitivity for mCHD under “research study” conditions. (31.6% × 58.1%/79.9%). The Specificity of the “routine reports” was assumed to be the same as the results under “research study” conditions (99.97%) (Additional file 1: Appendix VI).

For DL-US, our baseline screening sensitivity and specificity for sCHD were based on 95% and 96% respectively [73]. The baseline sensitivity and specificity of DL-US for mCHD were assumed to be the same as for”routine” US, 23.0% and 99.7% respectively. For POX screening at birth, the sensitivity and specificity for sCHD were 70.95% and 98.43% respectively (Additional file 1: Appendix VII).

### Healthy adjusted life expectancy (HALE)

The resultant discounted (and undiscounted) HALE for males with sCHD was 14.17 (24.2), 13.83 (23.4) and 13.78 (23.2) for prenatal diagnosis, diagnosis ≤ 24 h and diagnosis > 24 h. For sCHD females, the HALEs were 15.14 (27.3), 14.82 (26.5) and 14.79, (26.4) for prenatal, ≤ 24 h, > 24 h diagnoses respectively.

Due to their lower average lifetime disability weights (DWs) (0.061 vs 0.241 for sCHD), HALES were greater for mCHD. For males, the discounted (and undiscounted) HALEs were 20.87 (38.3), 22.81 (44.7) and 22.77 (44.6) for prenatal diagnosis, diagnosis ≤ 24 h and diagnosis > 24 h respectively. For mCHD females, HALEs were 21.92 (42.2), 23.62 (48.2), and 23.59 (48.06) for prenatal diagnosis, diagnosis ≤ 24 h and diagnosis > 24 h. HALE losses were calculated by subtracting these from the average populations discounted [and undiscounted] HALES of 29.66 [72.5] for males and 29.31 [73.0] for females [83].

### Cost utility ratios (CUR)

In our base line situation, the assumed higher sensitivity (95%) and lower specificity (96%) of DL-US (with and without POX) generated elevated usage of electrocardiograms and elective abortions, respectively. However, the effect of different interventions on miscarriages and stillbirths was minimal (Table 1). When no screening was undertaken (Additional file 1: Appendix VIII), the 905 sCHD fetuses that were viable at 12 weeks underwent 14 abortions, 87 miscarriages and 2 stillbirths, resulting in 802 live births with undiscovered sCHD (and similarly 1485 with mCHD). The use of only the US or POX alone led to 319 prenatal or 569 postnatal discoveries, respectively, of sCHD, resulting in 346 (48%) and 233 (29%) sCHD cases, respectively, being undiscovered before the infant was two days old (Additional file 1: Appendix IX). The current Israeli practice of screening by both US & POX, results in only 102 (or 15%) undiscovered cases out of 659 live births with sCHD (Additional file 1: Appendix VI). Use of DL-US has an expected higher sensitivity resulting in only 49 (8.7%) or 16 (2.9%) undiscovered cases with or without POX respectively.

**Table 1:** Inputs, Effects and Costs by Interventions type

	Null	US	POX	US	Deep	Deep
		Routine (a)		Routine & POX	Learning-US	Learning-US &POX
Echocardiogram (b)	0	672	3394	3742	8013	10,760
Ultrasounds	0	190,317	0	190,317	190,317	190,317
Abortions	2907	3084	2907	3084	5689	5689
Miscarriages	5637	5621	5637	5621	5610	5610
Stillbirths	567.2	566.7	567.2	566.7	566.4	566.4
POX tests	0	0	181,206	181,051	0	180,952
*COSTS: (million USD)*						
Intervention Costs	55.7	80.7	59.0	83.8	103.3	106.3
Treatment Costs	461.3	423.2	461.8	423.4	402.9	403.0

(a) Based on retrospective “routine reports”

(b) On true positive and false positive infants

Screening costs: US ($124.42), DL ($144.82)

DL Sensitivity (95%), Specificity (96%)

Due to its inherent influence on the learning process, DL-US likely to eventually have a higher specificity than US alone. However, if DL-US has a lower relative specificity, this would result in higher abortion rates (Additional file 1: Appendix VIII), which could cause the intervention costs of DL-US to be approximately 27% higher than those of US (Table 1), despite unit screening costs being only 6.1% higher [82]. Again, the increased sensitivity of DL-US results in lower QALY losses from CHD. These are offset by the increased QALY losses from abortions due to the possible lower specificity (Table 2). All interventions (except for POX) are both cost saving and add QALYs compared to doing nothing (“the null”)—that is, they “dominate” the null.

**Table 2:** QALYs gained, intervention and treatment costs by intervention

	Null	US	POX	US	Deep	Deep
		Routine (a)		Routine & POX	Learning-US	Learning-US & POX
Costs (million nis)						
Intervention Cost	55.7	80.7	59.0	83.8	103.3	106.3
Treatment Cost	461.3	423.2	461.8	423.4	402.9	403.0
Total Costs	517.0	503.9	520.8	507.2	506.2	509.3
Net Costs cf: null		− 13.1	3.8	− 9.8	− 11.8	− 7.7
QALY losses						
Mothers: Abortions	431.9	458.2	431.9	458.2	845.3	845.3
Mothers: Miscarriages	4088	4076	4088	4076	4069	4069
Mothers: Stillbirths	411	410.9	411.3	410.9	410.7	410.7
Mothers: Neonatal Mortality	91	47	43	27	22	19
Parents: due to Childs CHD	50	47	50	47	44	44
Patient: CHD lifetime	21,573	19,748	21,549	19,738	18,165	18,164
TOTAL QALY loss	26,646	24,787	26,573	24,756	23,556	23,552
QALYs gained cf null		1858	73	1889	** 3089**	3093
Average Cost-						
Effectiveness Ratio (b)		dom	50,882	dom	dom	dom
(USD per QALY gained)						

(a) based on retrospective “routine reports”

(b) dom: denotes intervention dominates the null by providing more QALYS at no additional cost

Screening costs: US ($124.42), DL ($144.82)

DL Sensitivity (95%), Specificity (96%)

POX, on its own costs approximately $51,000 per QALY (Table 2), deeming it to be marginally very cost-effective. The recent introduction of POX to prenatal US, increased costs by $3,304,000, and added 31 QALYs at a cost-effective incremental cost effectiveness ratio (ICER) of $106,600 per added QALY.

Substituting DL-US (& POX) for the current US protocol (& POX) would cost an extra $2.1 million but provide 1,204 more QALYs (Table 2) at a cost of $1,720 per QALY, which renders the intervention very cost-effective.

### Sensitivity analyses

Our one-way sensitivity analysis found the CUR to be insensitive (ie: inelastic, changing by < 10%) to a 10% change in treatment costs and QALY losses, both in total and especially in terms of their components (miscarriages, abortions, still births, neonatal mortality, parents and child’s lifetime QALY losses). A 10% increase of decrease in the relative cost of DL-US to US by 10% was found to have a far larger effect, quadrupling or decreasing the CUR by 77%.

By far the largest elasticity effects were related to the relatively unknown DL-US sensitivity and especially specificity for detecting sCHD, where 10% relative decreases elicited a tripling and a huge 17- fold increase in the CUR. Additionally, due to the paucity of information on DL screening characteristics we decided to present a table of feasible combinations of these DL characteristics, for three different cost- scenarios, relating to the meta-analyses of different US characteristics from routine operational and research studies. These tables provide ready- made estimates of CUR which can be referred to in the future, when more accurate DL characteristics become available. Meanwhile they give an indication as to the potential cost-effectiveness feasibility of adding DL (Table 3).

**Table 3:**
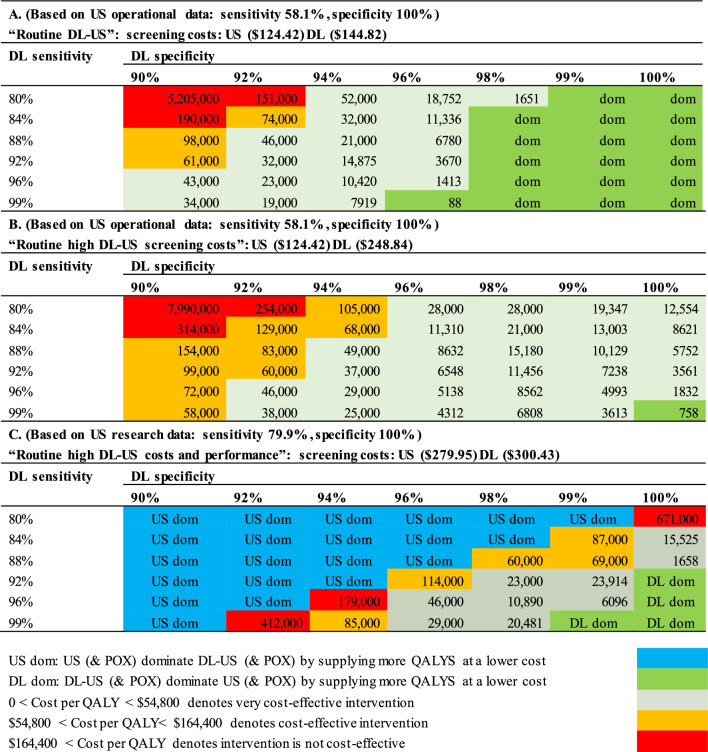
Costs per QALY (USD at 2022 price levels) of DL-US (& POX) vs US (& POX)

Based on data from the few “routine reports” on US that were based on actual real operational data, Option A (“Routine DL-US”), Table 3 A shows us where the advantage of DL-US (in terms of higher sensitivity) outweighs its possible disadvantage (due to possible lower specificities) versus US alone. Among all the combinations, where its sensitivity is > 94%, DL-US (& POX) is either very cost-effective by providing more QALYs at a relatively low extra cost (see Additional file 1: Appendix IX.A) or dominates US (& POX) by providing more QALYs at a lower cost.

In Scenario B (“Routine High DL-US Screening Cost”) where the price of DL-US was double that of US (Table 3B), DL-US (& POX) only dominated when the DL sensitivity was ≥ 99% and specificity was 100%. Despite their higher costs, DL (& POX) are still mainly cost-effective or very cost-effective (Additional file 1: Appendix IX.B).

In Scenario C (“Routine, High & Costly US Performance”), the relative advantage of DL-US is reduced, as it assumes greater achievements in the field of US screening efficiency levels attained under “research study” conditions. However, this higher US performance comes at a higher cost due to increased US screening time and supervision. US (& POX) dominates in many cells by providing additional QALYs at a lower cost (Table 3C). At higher specificity levels, cost-effectiveness and even very high cost-effectiveness are achievable by DL-US (& POX). Indeed, for some combinations (with a sensitivity and specificity of 99% and where a specificity level of 100% is accompanied by a sensitivity above 92%), DL-US (& POX) dominates US (& POX) because it is less expensive (Additional file 1: Appendix IX. C) in addition to providing more QALYs (Additional file 1: Appendix X.C.).

## Discussion

Our CUA focused on prospectively evaluating the anticipated substitution of DL-US for US in the future. The use of artificial intelligence-based DL-US in the diagnosis, risk stratification, and management of CHD is a promising future possibility given the current advancements in machine learning and knowledge of neural networks [57], paving the way for extremely efficient human error-free health care [84]. The evaluation of DL-US images is currently severely hampered by the lack of clinical trial data on the sensitivity and specificity of DL-US for identifying sCHD and mCHD. Expected gains in sensitivity (and subsequent survival of live births) will result in increases in the number of elective abortions.

If DL-assisted US screening is found to have a lower specificity than US alone, this might result in more voluntary abortions accompanied by fewer miscarriages and stillbirths. There is, however, currently no clear-cut evidence about the lower specificity of this tool, and if it is, it is likely to be corrected in the future as part of the learning process. This concern emphasizes the need to use this tool to support sonographer clinicians, who must have a final say in the diagnostic process.

Because of these limitations, we used our model to perform a range of sensitivity analyses, including some relating to an increased cost of DL-US screening to double that of US. Our study contributes to mapping out in advance the cost per QALY of various combinations of sensitivities and specificities, whose values are not yet known. Of course, oligopolistic suppliers of DL-US might use these data to increase DL-US costs up to the point where the intervention remains just cost-effective.

The most extensive meta-analysis of results from the “research study” perspective cannot overcome an inherent bias: that not only were the operators subject to more stringent quality controls of performance skills but also the time allocated to US performance (approximately 30 min) was greater than the 20 min devoted by busy community clinicians under “routine-reported” conditions. The potential comparative sensitivity advantage of DL-US compared to US increased (by 21.8% for sCHD and by 32.0% for mCHD) when US data were based on the three “routine” studies that were identified [69–71]. For this reason, in our baseline and first two analyses (Table 3A, B), we relied on data from “routine reports”.

“Routine reports” show greater resemblance to real-life routine practices than studies that are operated under prospective “research study” protocols. However, higher sensitivities have been reported in routine (reported) practices from a thoroughly organized national screening program with well-defined ultrasound protocols [17]. Therefore, the fact that someone cares, in routine practice, about quality control can provide an impetus toward better results. Uniform training and quality assessments of ultra sonographers within an integrated managed care consortium are additional factors for achieving greater sensitivities in both urban and rural areas [34].

The level of experience of the person performing or interpreting the scan [65, 85], as well as maternal characteristics [e.g., body mass index, abdominal scars] [3, 83, 86], affect the detection of foetal heart malformations. However, it is possible that the use of DL-US will ameliorate these problems. If this decrease occurs, then this will at least narrow the gap between DL-US sensitivities that will be reported under clinical trial and actual field conditions.

Our model included lifetime treatment costs and quality of life impacts, however we were only able to include social costs in the form of lost income from work of a parent as a result of miscarriages and still-births. Data on expected lifetime costs as a result of morbidity of newborns who survived with disabilities was not available. It is impossible to predict whether this would decrease of increase the CURs.

The option of primary prevention of CHD is unlikely to be feasible since 80% of CHD cases occur in foetuses of mothers without any risk factors [87, 88]. However, one should be open to exploring (via CUA) the feasibility of options such as adding additional US or DL-US screening in the second or third trimesters to mothers to be in any identified high-risk group. However, third-semester screening is unlikely to be cost effective due to the low incidence and severity of detectable defects [89, 90].

The decision to recommend adding POX to the existing US protocol was made without any ex-ante cost-utility analysis based on an Israeli setting. Cost-effectiveness analyses from other countries resulted in decisions to implement POX (i.e., Israel was in comparative need of this intervention), in addition to the logical assumption that the benefits of postnatal diagnoses via POX can be achieved at a very low cost. It should be noted that in some of the other countries, evaluative studies of POX did not even factor in the cost of nursing due to the short time needed to complete the screening [42]. Indeed, our retrospective (ex-post) CUA showed the original decision to add POX to be cost-effective and correct from a health economic viewpoint. The diffusion of this cheap technology appears to be far faster than was initially anticipated [91]. Following the national policy decision to adopt the technology in 2021, a recent survey reported that it had been implemented by all Israeli hospitals in 2023.

However, hospitals that have implemented POX screening have been reported to be able to do so using existing nursing staff and do not incur additional staff costs. From the hospital perspective, the cost of staff time need not be included. From a societal perspective, the inclusion of staff time makes sense if the nursing time used for POX screening could have been used for other tasks. If nursing time could not be reallocated, the fact that our estimates included a costing of nursing time would cause an overestimation of the CUR for POX screening [92].

Falling outside the domain of this paper are machine learning algorithms, which include the perfusion index, heart rate, pulse delay and photoplethysmography characteristics; these algorithms have been reported to improve the sensitivity of cCHD detection by ten percentage points over pulse oximetry screening alone [93].

The calibration and structure of the model were constrained by the availability of the data. Unfortunately, for CHD patients diagnosed > 24 h after birth, no mortality, QALY or cost data have been published by age (in weeks or months) at CHD discovery. The delayed discovery of CHD associated with pulmonary hypertension and increased neurodevelopmental morbidity may lead to higher lifetime treatment costs and undesirably higher mortality rates. Early diagnosis and treatment can reduce the incidence of irreversible and intractable pulmonary hypertension through its associated morbidity, treatment costs and complications. The availability of such data would have enabled us to calculate the cost-effectiveness of adding additional screening strategies after the infant is discharged from the hospital.

The impact of disease on families of patients has often gone unrecognized and is therefore underestimated [94]. Measurements of the impact are usually disease specific [94] and have been expressed only in very rare instances in utility values, such as the caregiver burden of spouses with dementia [95]. Therefore, we attempted to estimate the impact of CHD on the quality of life of one (for single parents) or both parents.

An Egyptian study reported that parents of children with heart disease scored worse on QOL scales in all dimensions except bodily pain [96]. Mothers have been reported to have greater stress [97] and to report feelings of anger, sadness, loneliness, helplessness, numbness, and confusion [98]. In contrast to one study [53] in which QALY loss was ceased from the mother’s perspective after her death, we applied these values to the child over the child’s expected lifetime.

We also added the expected QALY losses of the father (if present), who is more likely to report feelings of shock, such as when first learning about the diagnosis at the postnatal stage, treatment plan or unexpected complications [98]. Fathers often described their stress as not being able to protect their infant from CHD and from difficulties balancing employment (despite coworker support and being allowed flexible scheduling) with support for their partner and care of their child when hospitalized [98].

A prospective longitudinal study [99] [based on the Assessment of Quality of Life (AQoL)-8D Multi-Attribute Utility Instrument [100]] of the quality of life in parents of seriously Ill/injured children hospitalized in cardiology, oncology or intensive care wards was performed. The study reported decreased quality of life (compared with that of parents of healthy children) of 0.0376 and 0.0048 after four weeks and seven months, respectively. The figure for four weeks was close to the 0.03 loss we used in our model based on parents of CHD children. If the WHO DWs that we used for child and adult CHD were based on parental valuations, then these are likely to have under-estimated the DWs as felt by the child or adult with CHD [101].

For both the few “routine reports” and the many “research study” reports, the data were extracted from a recent meta-analysis of first trimester screening [82]. Despite a great deal of caution used in the estimation of false positives [82], there is a possibility that specificities were overestimated, leading to underestimates of the potential for improvement by adopting DL and hence upwardly biased CUA ratios.

Other factors that caused an upwards bias in our cost-utility analysis (towards higher costs per QALY) include the following:

i) We excluded parental QOL losses on account of children who were aged 18 and older. QOL losses are especially likely to still occur in the parents of young adults with sCHD.
ii) We did not attempt to estimate the impact on the quality of life of siblings [102, 103] or members of the extended family [94], especially grandparents.
iii) Our perspective did not include work losses, transport costs, out-of-pocket expenses resulting from the screening or premature burial costs.
iv) A prenatal diagnosis has been found to increase the level of parental distress from diagnosis to six months after birth [104]. We did not impute the QOL effects of parental worry from fetal diagnosis (or misdiagnosis) until abortion, mis-carriage or birth.
v) If the WHO DWs for CHD that we used were based on parental valuations, then these DWs are likely to have underestimated the DWs as felt by the child or adult with CHD [40].
vi) The added costs of litigation in connection with CHD were not included. These include not only the direct costs of litigation (such as lawyers and possible court costs) but also increased insurance premiums, defensiveness reactions and burn-out from misdiagnoses in the current adversarial legal system.
vii) One of the three “routine reports”, was carried out in a high-quality setting, with physician performed US taking a long time (approximately 30 min) with additional (transvaginal) views as required [69]. This results in underestimation of the potential for improvement by adopting DL-US.

Factors causing a downwards bias in our cost‒utility analysis (towards lower costs per QALYs) include the following:

i) If the WHO DWs for CHD were based on health professionals’ valuations, then these DWs are likely to have over-estimated the DWs as felt by the child or adult with CHD [101].
ii) The extent to which CHD treatment costs that were associated with conditions might be underestimated in our model. Lifetime CHD disease-specific costing is essential for improving these estimates.
iii) Clearly not all persons losing a pregnancy due to miscarriage, stillbirth or abortion would try to replace their loss by having another pregnancy.

A factor whose direction of bias is unknown is that we did not account for the impact of a false-positive CHD diagnosis because the effect of the initial parental stress is hard to quantify (an additional question can be asked if the mothers’ stress could affect the foetus) and is offset partly or more than totally by the relief obtained once patients learn that the foetus is indeed unaffected. However, given the very high specificity of both initial heart screening (US or DL-US) and confirmation by foetal echocardiography, the number of pregnant women (and indeed their spouses) experiencing this issue would be rather small [53].

Because of lack of available data, our analysis was unable to model cost-saving and improved outcomes by DL-US related early CHD detection and prevention of irreversible and/or intractable pulmonary hypertension. We failed to find literature data with separation of outcomes for CHD detected at birth from those diagnosed several months or years later (leading us to include detections at > 24 h as one variable). Since late diagnosis incurs high mortality and costly morbidity—including permanent neurodevelopmental defects, it is likely that incorporating this issue in CUA would have made the adoption of DL-US even more advantageous.

The adoption of DL-US can improve health systems not only in the administrative (e.g., eligibility) and operational (e.g., operating room and ER management) domains but also in the clinical domain [92]. We believe that even early analyses (i.e., before all DL-US performance information is available), such as those we have undertaken, can accelerate the adoption of this new technology.

Unless there is a substantial decrease in relative specificity, the increase in clinical sensitivity provides a great impetus for the adoption of DL-US. Our exploratory CUA calculations point to the possibility of DL-US being cost-effective, despite the weakness of the data in that they were not based on screening characteristics from meta-analyses of clinical trials using DL-US.

### Supplementary Information

**Additional file 1: **Appendix Ia: Major Diagnostic Modes. Appendix Ib: Major Diagnostic Modes. Appendix Ic: Major Diagnostic Modes. References to Appendices 1a,1b & 1c.

## Data Availability

Data sharing is not applicable to this article, as no datasets were generated or analysed during the current study. Spreadsheet calculations that support the findings of this study are available upon reasonable request from the corresponding author.

## Abbreviations

ACER: Average cost effectiveness ratio
AQoL: Assessment of quality of life
cCHD: Critical congenital heart disease
mCHD: Minor congenital heart disease
sCHD: Serious congenital heart disease
CHD: Congenital heart disease
CUR: Cost utility ratio
DL: Deep learning
DW: Disability weight
GDP: Gross domestic product
HALE: Healthy adjusted life expectancy
HLHS: Hypoplastic left heart syndrome
ICER: Incremental cost effectiveness ratio
NIS: New Israel Shekles
POX: Pulse oximetry
QALY: Quality adjusted life year
QOL: Quality of life
TGA: Transposition of great arteries
UFE: Universal fetal electrocardiograpic
US: Ultrasound
USD: United States Dollars
