# Supplementary material for: Cost-utility analysis of prenatal diagnosis of congenital cardiac diseases using deep learning
Source: Cost Eff Resour Alloc. 2024 May 22;22:44. doi: 10.1186/s12962-024-00550-3 (PMC11110271; doi:10.1186/s12962-024-00550-3)
Supplement: Supplementary file 1 — Additional file 1: Appendix Ia: Major Diagnostic Modes. Appendix Ib: Major Diagnostic Modes. Appendix Ic: Major Diagnostic Modes. References to Appendices 1a,1b & 1c. [file 12962_2024_550_MOESM1_ESM.docx]

**Appendix Ia: Major Diagnostic Modes**

**Ultrasound (US)**

Over the years, improvement of obstetric ultrasound (US) is attributed to many factors, including developments in education, accreditation, guidelines, quality assurance, anatomical and physiological knowledge, and imaging quality (1).

There is however, a large difference between sensitivities attained under optimal (usually “research study” conditions) and routine everyday practices. Optimal US results were by and large generated by referral institutions affiliated with academic centers often under prospective conditions.   Far lower US sensitivities were reported from a few studies (2-4) from routine, overloaded community-based practices (5) that allocated far less time to the screening than under optimal conditions. Thus, the potential for improvement and the incentive to improve post-natal screenings (see pulse oximetry and deep learning below) is, in reality, far greater than what has been reported in the literature dominated by research studies with high quality control conditions.

In Israel, until 2023, routine screening for both sCHDs and mCHDs relied on a mid-trimester anomaly scan in pregnant women, involving basic sectional imaging of heart anatomy and function as well as a postnatal auscultation by stethoscope (6). US detection rates show a strong correlation with CHD severity (7). However, like some other countries, there is no required accreditation for US in Israel despite the evidence that the lack of accreditation is highly correlated with poorer performance quality (8,9). This results in US sensitivity in Israel falling still further below the sensitivities reported in optimal prospective trials and in retrospective studies (especially for mCHD), resulting in many newborns being discharged from hospital before CHD was diagnosed (6).

Even some members, though by no means all, of specific groups, (ultra-orthodox Jews and Muslims) who are strictly opposed to aborting even severely malformed foetuses (10), opt to undergo an US scan for anomalies, which can at least give them time to prepare for a possibly challenged infant.

Postnatal echocardiography is the established gold standard for diagnosing CHDs. However, echocardiography may also contribute to an apparent rising incidence of CHDs mainly as a result of the detection of abnormalities which are of no functional or clinical significance (11,12). As a result, echocardiography is likely to have significant limitations as a screening tool, not only due to elevated false positive rates (13,14), but also as a result of cost and qualified manpower constraints (6).

**Appendix Ib: Major Diagnostic Modes**

**Pulse Oximetry (POX)**

Infants not detected with sCHD before discharge leaving hospital had far lower hospitalization days and hospital costs during infancy (up to 12 months old) than persons with timely detection between birth and hospital discharge. However, after adjusting for mothers age, race, education, payor status and the infants gender gestation period and type of sCHD, a 64% reduction in costs was changed to a 35% increase in relative costs for the undetected group, as a result of the groups 52% higher hospital admission rate and 18% higher utilization of hospitalisation days (15).

In the USA, statewide implementation of mandatory policies for newborn screening for critical CHD was associated with a significant decrease in infant cardiac deaths between 2007 and 2013 compared with states without these policies (16).

A policy of universal pulse oximetry survey in newborns was rejected in 2013 by the National Council for Child Health and Pediatrics in Israel because of the argument that, in this country, an ultrasound is nearly always performed – and with the mistaken assumption that its sensitivity is high. Following the tragic case of the child – to whom this paper is dedicated – a national policy was adopted in 2021 for pulse oximetry (POX) in newborns and a committee of experts was appointed to advise about quality control in pregnancy US with special consideration for Artificial Intelligence (AI) implementation in this field.

POX, measures the percentage of haemoglobin saturated with oxygen (17) in a simple, quick, safe, painless (6), non-invasive way (18) that is acceptable to both parents and staff (6). In addition, POX may also prove to be beneficial in conditions that cannot be identified before birth, including coarctation of the aorta that occurs with duct closure – 48 hours after birth.

Besides not being distressing for the baby, and reassuring for parents. POX has the potential to detect problems soon after birth, before discharge, allowing treatment to be started and lives to be saved (22). POX levels usually below 95% [subject to adjustment by altitude (10,19)] indicate possible sCHD in the new-born. Those not achieving predetermined oxygen saturation thresholds are usually referred for echocardiography (6).

In a national survey, around 78% of the neonatal units in the UK that used POX, responded that they felt that screening did not lead to an increase in the number of unnecessary investigations, while 10% of the neonatal units felt that any small increase was justified and offset by the benefits of identifying considerable cardiac and non-cardiac pathology (20).

However, even the combined sensitivity of US (and POX) is insufficient (21,22) and a significant proportion of both sCHD and mCHD are diagnosed post-discharge (23).

**Appendix Ic: Major Diagnostic Modes**

**Deep Learning & Ultrasound (DL-US)**

It is hoped that in the future, the evolution of deep learning (DL) based on artificial intelligence (AI) and data science, will be integrated into mechanizing several aspects of medical care requiring critical thinking: including diagnosis, risk stratification, and management, thus reducing both physician’s burden and the likelihood of human error. The use of neural networks and machine learning may significantly improve the diagnostic value (24) of cardiac magnetic resonance imaging, echocardiograms, computer tomography scans and electrocardiographs, in consequence augmenting and improving the diagnostic accuracy of detecting foetal CHD (25-28).

AI models have been found to be statistically superior to standard foetal biometry–based gestational age estimates derived from images captured by expert sonographers in estimating gestational age (29). AI applied to ultrasound examination of the foetal heart has been explored to improve diagnostic accuracy in the context of foetal CHD (24,26,30), matching “expert” performance levels (26). However, there is a paucity of information about the impact that AI might have on national screening for CHD. Another study reported that electrocardiograms using DL-US outperformed diagnostically (i.e. greater sensitivity and specificity), cardiologists reading of electrocardiograms for atrial septal defects (31).

DL-US has the potential to level the playing field for centers that obtain access to this technology. Particularly for those hospitals that are exposed to a low volume of CHD and/or have sonographers with limited exposure to and training on fetal CHD. Also, for those in populations with a lower socioeconomic status, whose maternal rates of pre-existing diabetes additionally puts their patients at higher risk for having a child with CHD (32).

**References to Appendices 1a,1b & 1c.**

1. Drukker L, Sharma H, Karim N, Droste R, Noble JA, Papageorghiou AT. Clinical workflow of sonographers performing fetal anomaly ultrasound scans: deep-learning-based analysis, Ultrasound Obstet Gynecol 2022;60:759-65.

2. Syngelaki A, Hammami A, Bower D, Zidere V, Akolekar R, Nicolaides KH. Diagnosis of fetal non-chromosomal abnormalities on routine ultrasound examination at 11–13 weeks’ gestation. Ultrasound Obstet Gynecol 2019; 54: 468–76.

3. Andrew C, Gopal S, Ramachandran H, Suvika M. First Trimester Ultrasound: Addition of Anatomical Screening Adds Value to the Examination: A Retrospective Case Series Journal of Evolution of Medical and Dental Sciences 2015; 4: 2690-2699.

4. Vayna AM, Veduta A, Duta S, Panaitescu AM, Stoica S, BuinoiuN, et al. Diagnosis of Fetal Structural Anomalies at 11 to 14 Weeks. J Ultrasound Med 2018; 37:2063–73.

5. Krishnan A, Donofrio MT. Impact of Socioeconomic Status, Race and Ethnicity, and Geography on Prenatal Detection of Hypoplastic Left Heart Syndrome and Transposition of the Great Arteries. Circulation 2021;143:2049–60.

6. Ewer AK, Furmston AT, Middleton LJ, Deeks JJ, Daniels JP, Pattison HM et al.

Pulse oximetry as a screening test for congenital heart defects in newborn infants:

a test accuracy study with evaluation of acceptability and cost-effectiveness. Health Technol Assess 2012;16: 2::v-xiii, 1-184.

7. van Velzen CL, Ket JCF, van de Ven PM, Blom NA, Haak MC. Systematic review and meta-analysis of the performance of second-trimester screening for prenatal detection of congenital heart defects.

Int J Gynecol Obstet 2018; 140: 137–45.

8. Sorrentino K. Accreditation, Credentialing, and Quality Improvement in Diagnostic Medical Sonography: A Literature Review. Journal of Diagnostic Medical Sonography 2019; 35: 401–11.

9. Levy DJ, Pretorius DH, Rothman A, Gonzales M, Rao C, Nunes ME et al. Improved Prenatal Detection of Congenital Heart Disease in an Integrated Health Care System. Pediatr Cardiol 2013;34:670–9.

10. Bin-Nun A, Hammerman C, Mimouni FB, Wasserteil N, Kasirer YM. The Saga of Pulse Oximetry Screening for Critical Congenital Heart Disease in Israel: A Historical Perspective. IMAJ 2021; 23: 229-32.

11. Hoffman JIE, Kaplan S. The incidence of congenital heart disease. J Am Coll Cardiol 2002;39:1890–900.

12. Wren C, Richmond S, Donaldson L. Temporal variability in birth prevalence of cardiovascular malformations. Heart 2000;83:414–19.

13. Knowles R, Griebsch I, Dezateux C, Brown J, Bull C, Wren C. Newborn screening for congenital heart defects: a systematic review and cost-effectiveness analysis. Health Technol Assess 2005; 9(44):1-152, iii-iv.

14. Mahle WT, Newburger JW, Matherne GP, Smith FC, Hoke TR, Koppel R, et al. Role of pulse oximetry in examining newborns for congenital heart disease: a scientific statement from the AHA and AAP. Pediatrics 2009;124:823–36

15. Peterson C, Dawson A, Grosse SD, Riehle-Colarusso T, Olney RS, Tanner JP, et al. Hospitalizations, costs, and mortality among infants with critical congenital heart disease: how important is timely detection? Birth Defects Research. Part A, Clinical and molecular teratology. 2013;97:664-72.

16. Abouk R, Grosse SD, Ailes EC, Oster ME. Association of US State Implementation of Newborn Screening Policies for Critical Congenital Heart Disease With Early Infant Cardiac Deaths JAMA. 2017;318:2111-8.

17. Plana_MN, Zamora_J, Suresh G, Fernandez-Pineda_L, Thangaratinam_S, Ewer_AK. Pulse oximetry screening for critical congenital heart defects. Cochrane Database of Systematic Reviews 2018, Issue 3. Art. No.: CD011912. DOI: 10.1002/14651858.CD011912.pub2.

18. Roberts TE, Barton PM, Auguste PE, Middleton LJ, Furmston AT, Ewer AK. Pulse oximetry as a screening test for congenital heart defects in newborn infants: a cost-effectiveness analysis, Arch Dis Child 2012;97:221–6.

19. Guo F, Tang S, Guo T, Bartell S, Detrano R. Revised threshold values for neonatal oxygen saturation at mild and moderate altitudes. Acta Paediatr 2020; 109:2: 321-6

20. Brown S, Liyanage S, Mikrou P, Singh A, Ewer AK. Newborn pulse oximetry screening in the UK: a 2020 survey. The Lancet September 26, 2020; 396;881.

[21. Campbell MJ, Quarshie WO, Faerber J, Goldberg DJ, Mascio CE, Blinder J. Pulse Oximetry Screening Has Not Changed Timing of Diagnosis or Mortality of Critical Congenital Heart Disease Pediatr Cardiol. 2020 Jun;41:899-904..](https://pubmed.ncbi.nlm.nih.gov/32107587/)

22. Chakraborty A, Gorla SR, Swaminathan S. Impact of prenatal diagnosis of complex congenital heart disease on neonatal and infant morbidity and mortality Prenatal Diagnosis 2018;38: 958–63

23. Bonnet D. Impacts of prenatal diagnosis of congenital heart diseases on outcomes Transl Pediatr 2021;10(8):2241-49.

24. Day TG, Kainz B, Hajnal J, Razavi R, Simpson JM. Artificial intelligence, fetal echocardiography,

and congenital heart disease. Prenatal Diagnosis 2021;41:733–42.

25. Sethi Y, Patel N, Kaka N, Desai A, Kaiwan O, Sheth M et al. Artificial Intelligence in Pediatric Cardiology: A Scoping Review. J. Clin. Med 2022; 11: 7072

26. Arnaout R, Curran L, Zhao Y, Levine JC, Chinn E, Moon-Grady AJ. An ensemble of neural networks provides expert-level prenatal detection of complex congenital heart disease. Nature Medicine 2021; 27: 882–91.

27. Carroll AE, Downs SM. Improving decision analyses: parent preferences (utility values) for pediatric health outcomes. J Pediatr 2009; 155: 21–5.

28. Day TG, Kainz B, Hajnal J, Razavi R, Simpson JM. Artificial intelligence, fetal echocardiography,

and congenital heart disease. Prenat Diagn 2021; 41: 733–42.

29. Lee C, Willis A, Chen C, Sieniek M, Watters A, Stetson B et al. Development of a Machine Learning Model for Sonographic Assessment of Gestational Age. JAMA Network Open 2023;6:1:e2248685.

30. Garcia-Canadilla P, Sanchez-Martinez S, Crispi F, Bijnens B. Machine Learning in Fetal Cardiology: What to Expect. Fetal Diagn Ther 2019 : 149–53.

31. Mori H, Inai K, Sugiyama H, Muragaki Y. Diagnosing Atrial Septal Defect from Electrocardiogram with Deep Learning. Pediatric Cardiology 2021; 42:1379–87.

32. Morris SA and Lopez KN. Deep learning for detecting congenital heart disease in the fetus. Nature Medicine 2021;27; 759–65.

**Appendix II. Modelling Survival**

Modelling possible survival gains due to pre or timely post-natal diagnosis is particularly complex. Comparing one-year survival rate among those with noncritical CHDs alone (n = 2,455) showed no difference between prenatal and postnatal diagnoses (96% vs 98%, respectively, p = 0.26), whereas among those with critical CHDs (n = 691), prenatally diagnosed infants had significantly lower survival rate, 71% vs 86%, respectively (1).

Among infants with critical CHDs, the adjusted hazard ratio for one -year mortality of those prenatally versus postnatally diagnosed was 2.51 (95% CI 1.72 to 3.66). Prenatal diagnosis was associated with lower one-year survival rates for infants with isolated critical CHDs but showed no change for those with isolated noncritical CHDs. The precise explanation as to why those whose critical CHDs are diagnosed earlier seem to have poorer survival is likely a reflection of the severity of disease, i.e. those that have more severe disease (even within one diagnostic category) are more likely to be diagnosed earlier and are also more likely to have poorer survival. (1-3).

In a similar way, severe diagnoses are more likely to be discovered during the first 24 hours after birth (as opposed to > 24 hours) and therefore can explain the higher infant mortality rate (82.5% vs 71.7% in babies diagnosed after 24 hours) (4). A similar gradient is found in that infants diagnosed after hospital discharge had lower mortality rates than those diagnosed before discharge, who in turn had lower mortality rates than those with a prenatal diagnosis (5).

The superiority of a prenatal diagnosis manifests itself in allowing surgical procedures to be carried out in the early neonatal period (6). When high-risk infants and comfort care infants are excluded, infants with a prenatal diagnosis had far lower pre-operational mortality rates. However, there were more high-risk and comfort-care patients in the prenatal compared with the postnatal diagnosis group (7). One cost-effectiveness analysis was driven by experts’ opinion that assumed a 20% higher mortality rate for diagnoses based on the post- as opposed to pre- natal stages (8).

Early diagnosis of CHD is infants is imperative since delayed diagnosis of congenital heart disease worsens the preoperative condition and outcome of surgery in neonates (9). However, survival was found to be lower in those who were diagnosed prenatally than postnatally (10). The explanation being that the more severe defects (with higher mortality rates) are easier to diagnose and consequently minor defects (with lower mortality rates) are harder to diagnose. Therefore, we attempted to control for the diagnosis in order to measure the benefits of diagnosing early (eg: prenatally or in the first day of life). However, even after controlling for diagnosis, more severe cases of the same diagnosis are still be more likely to be discovered before less severe cases, thereby being prone to worst outcomes (11). This phenomena is evidenced by a study (11) that reported lower infant survival rates for prenatal diagnosis (compared to postnatal diagnosis) for Single Ventricular [SV] (46.9% vs 57.1%) and d-transposition of the great arteries (TGA) (91.2% vs 95.8%). However, the study (12) also reported similar survival rates for COA (93.1% vs 93.2%) and non-significant improved survival for TOF (97.2% vs 88.9%).

When analyses are diagnosis-specific there is less (though not zero) selection bias of easily detectable severe cases at the prenatal stage. Overall, after taking into account, those who refused surgery, there was a higher survival rate (63.6% vs 45.5%) in live births, that had a prenatal diagnosis of HLHS (13). Another study reported that prenatal diagnosis resulted in remarkably reduced the pre-operative (22%, 95% CI 6% - 80%) and post-operative mortality (11%, 95% CI 1% - 83%) rates in cases (6) with TGA.

A similar advantage to prenatal screening when a group of subtypes (consisting of TGA, HLHS, SV, TOF and double outlet right ventricle (DORV)) with reductions in the pre-operative (41%, 95% CI 18% - 94%) and post-operative mortality (66%, 95% CI 46% - 94%) rates. In India, prenatal diagnosis, and planned delivery of neonates with critical CHD was associated with significantly lower costs of cardiac care (14). In parallel, reduced severity when controlling for diagnosis was reflected in a retrospective study of infants with TGA from the USA, that reported 22% higher hospitalization costs in infants without a prenatal diagnosis (15).

**References to Modelling Survival**

1. Oster ME, Kim CH, Kusano AS, Cragan JD, Dressler P, Hales AR et al. A Population-Based Study of the Association of Prenatal Diagnosis With Survival Rate for Infants With Congenital Heart Defects. Am J Cardiol. 2014; 113:1036–40.

2. Bak GS, Shaffer BL, Madriago E, Allen A, Kelly B, Caughey AB et al. Detection of fetal cardiac anomalies: cost-effectiveness of increased number of cardiac views. Ultrasound Obstet Gynecol 2020; 55: 758–67

3. Han B, Tang Y, Qu X, Deng C, Wang X, Li J. Comparison of the 1-year survival rate in infants with congenital heart disease diagnosed by prenatal and postnatal ultrasound: a retrospective study. Medicine 2021;100:4 (e23325).

4. Oster MR, Lee KA, Honein MA, Riehle-Colarusso T, Shin M, Correa A. Temporal Trends in Survival Among Infants With Critical Congenital Heart Defects Pediatrics 2013 ; 131 :5: e1502–8.

5. Brown KL, Ridout DA, Hoskote A, Verhulst L, Ricci M, Bull C. Delayed diagnosis of congenital heart disease worsens preoperative condition and outcome of surgery in neonates. Heart 2006;92:1298–1302.

6. Li Y-F, Zhou K-Y, Fang J, Wang C, Hua Y-M, Mu D-Z. Efficacy of prenatal diagnosis of major congenital heart disease on perinatal management and perioperative mortality: a meta-analysis. World J Pediatr 2016; 12: 298-307.

7. Holland BJ, Myers JA, Woods CR, Prenatal diagnosis of critical congenital heart disease reduces risk of death from cardiovascular compromise prior to planned neonatal cardiac surgery: a meta-analysis. Ultrasound Obstet Gynecol 2015; 45: 631–8.

8. Mukerji A, Shafey A, Jain A, Cohen E, Shah PS, Sander B,Shah V. Pulse oximetry screening for critical congenital heart defects in Ontario, Canada: a cost-effectiveness analysis. Canadian Journal of Public Health 2020; 111:804–11.

9. Brown KL, Ridout DA, Hoskote A, Verhulst L, Ricci M, Bull C. Delayed diagnosis of congenital heart disease worsens preoperative condition and outcome of surgery in neonates. Heart 2006;92:1298–1302.

10. Han B, Tang Y, Qu X, Deng C, Wang X, Li J. Comparison of the 1-year survival rate in infants with congenital heart disease diagnosed by prenatal and postnatal ultrasound: a retrospective study. Medicine 2021;100:4(e23325).

11. Wright LK, Ehrlich A, Stauffer N, Samai C, Kogon B, Oster ME. Relation of Prenatal Diagnosis With One-Year Survival Rate for Infants With Congenital Heart Disease. Am J Cardiol 2014;113:1041e1044,

12. Khoshnood B, Lelong N, Houyel L, Bonnet D, Ballon M, Jouannic J-M et al Impact of prenatal diagnosis on survival of newborns with four congenital heart defects: a prospective, population-based cohort study in France (the EPICARD Study). BMJ Open 2017;7:e018285.

13 Tworetzky W, McElhinney DB, Reddy VM, Brook MM, Hanley FL. Silverman NH, Bristow J. Improved surgical outcome after fetal diagnosis of hypoplastic left heart syndrome. Circulation 2001; 103:1269-73.

14. Vaidyanathan B, Rani K, Kunde F, Thomas S, Sudhakar A, Kumar RK, Zheleva B. Prenatal diagnosis lowers neonatal cardiac care costs in resource-limited settings. Cardiology in the Young 2022; 32: 1754–60.

15. Gupta D, Mowitz ME, Lopez‐Colon D, Nixon CS, Vyas HV, Co‐Vu JG. Effect of prenatal diagnosis on hospital costs in complete transposition of the great arteries. Prenatal Diagnosis. 2018;38: 67–571.

**Appendix III: Methodology of Calculating the Cost-Utility ratios by Screening Interventions.**

In all our supporting meta-analyses, we used a dynamic search methodology, where to take the example of survival:- initially 36 articles were identified (using search terms “prenatal diagnosis’ AND “postnatal diagnosis” AND (survival or mortality) AND “congenital heart”). Of these, only seven “core” articles (1-7) had some relevant information on diagnosis specific survival differences. Next, searches made not only on references in the core articles, but also by means of searching PubMED for later articles that referenced the core article. The newly identified references were in turn used to identify further articles. This process was repeated until no new relevant articles were found. A similar dynamic method was used to construct the meta-analyses of sensitivities and specificities of US and POX screening as well as miscarriage and abortion rates.

The back calculation of the null (no intervention scenario) and expected births by CHD status (serious, minor, or non-CHD) for each intervention involved the following steps.

a) The 162,489 births in the mid-year of the 2005-2014 period (referred to as its mid-point years 2010/11), multiplied by the specific incidence rates of 3.42 and 1.147 per 1000 for serious and minor CHD respectively (8), in order to estimate the numbers of live births by CHD status (serious, minor, and non-CHD).

b) CHD specific miscarriage rates of 9.71% (Appendix XI) and 3.14% (Appendix XII) for serious and minor CHD respectively were derived from meta-analyses of the literature. Based on an estimated overall post - first trimester 3% miscarriage rate (9), an estimate of 2.97% was made for non-CHD fetuses.

c) CHD specific stillbirth rates (1.010%, 0.327% and 0.309% for serious, minor and non-CHD were estimated by applying the overall 0.312% stillbirth rate (in 2010/11), the mid - point of the 2005-2014 era) (10) in proportion to the CHD specific miscarriage rates.

d) The number of fetuses viable after terminations of pregnancies (TOPs or abortions) related to first semester ultrasounds were (back-) calculated based on applying still birth (11) and miscarriage rates (9) to the live birth rates.

e) Abortion data from 2019 (12,13) was applied to livebirth data (10) to estimate the number of abortions that were actually performed in 2011 by CHD status.

f) The actual number of fetuses viable before week 13 by CHD status was calculated by adding the abortion data (e) to the fetal data (d).

g) The number of fetuses by CHD status undergoing ultrasound was estimated by applying the assumed 99% percentage of pregnant women undergoing first trimester ultrasound to the fetal numbers in f).

h) For fetuses undergoing ultrasound, the sensitivity (58.7% and 19.9%) and specificities (99.991% and 99.995%) of sCHD (Appendix XIII) and mCHD (Appendix XIV) first trimester ultrasounds respectively (based on a meta-analysis of publications in the 2005-2014 era) were applied to the fetal numbers (g) and the numbers aborting (e). These provided estimates of 32.4%, 11.6% and 1.5% abortion rates (based on the sum of true positives and false positives) for the for serious, minor, and non-CHD categories respectively.

i) Next the abortion, miscarriage and stillbirth rates were applied to those who had undergone ultrasound in order to estimate the live births by CHD status.

j) Only the miscarriage and stillbirth rates were applied to the 1% who did not undergo ultrasound to estimate the live births by CHD status.

k) The data from 2005-14 was applied to the updated (by means of a meta-analysis based on the 2015-2022 period) ultrasound sensitivity of 71.3% and 15.5% and specificity of 99.986% and 99.992% for sCHD (Appendix V) and mCHD (Appendix VI) respectively. The addition of abortion, miscarriage and still birth rates were then used to estimate the CHD status at birth in 2022.

l) Next, the data in k) was adjusted backwards to a situation where no woman undertook an ultrasound, this is in effect the null (do nothing) scenario which formed the basis for future calculations.

m) The following interventions were applied in turn to the null scenario in order to generate estimates of abortions, miscarriages, stillbirths, and CHD specific estimates (serious, minor, or non-CHD) livebirths:

i) First trimester ultrasound based on sensitivity and specificity derived from a meta-analysis of publications (with and without POX).

ii) First trimester ultrasound using deep learning AI based on a preliminary study on sCHD that provided sensitivity and specificity (14) estimates of 98% and 96% respectively (with and without POX). A sensitivity analysis explored parameters for DL for detecting both sCHD and mCHD.

iii) Use of POX alone, was based on a meta-analysis (Appendix VII) of publications from 2000-2022 [including those identified in a supplement to a Canadian study (15)] where the cutoff point was 95% that did not exclude fetuses that had already obtained positive fetal diagnoses from prenatal ultrasounds.

n) Disease specific one year survival rates by prenatal or postnatal diagnosis (see Appendix II) were based on meta-analyses of the post 2000 literature (see Supplementary Materials). In order to control for diagnoses, the percentage prevalence of sCHD for each specific diagnosis in Israel (8) was used to calculate weighted average specific mortality rates (for prenatal and postnatal diagnoses).

o) Using data on the comparative survival of infants diagnosed <= and > 48 hours after birth (10), the postnatal survival rate for sCHD was decomposed into assumed <= and >24 hours after birth rates in order to capture any relative advantage of POX screening during the first day of life.

p) Survival of mCHD infants by prenatal and postnatal diagnosis was based on a single study (16), with the postnatal rates again adjusted into pre- and post- 24-hour diagnoses.

q) The one-year survival rates were extended (Appendix XV) by integrating meta-analysis data on 1,5,10 and 15 years survival (17) by diagnosis weighted by prevalence rates from an Israeli study (18) for mCHD and by prevalence rates from a national study (8) for sCHD.

r) Our model estimated neonatal mortality rates for sCHD, mCHD and infants without CHD of 116, 23 and 1.2 per 1000 births (11) respectively, which were used in the calculation of maternal QALY losses.

s) National age and gender specific mortality rates (11) were applied to the relative survival rates for 15 year -olds by (CHD type and time of diagnosis) to estimate survival rates for 16-19 years old’s and were applied to the relative infant mortality survival rates to estimate survival rates for 20-99 years olds.

**Treatment Costs**

t) The percentage of sCHD with morbidity and treatment costs by age groups [0 and1-17] were obtained for sCHD (with and without morbidity) based on Canadian 2010/11 price levels (15). These were linked using the Canadian price index and then converted to Israeli shekels in 2022 by the purchasing power parity exchange rate for the 75% of costs were assumed to be non-tradeable services (eg: salaries) and by the exchange rate for the remainder of any tradeable goods.

u) Recent Israeli (18) age group specific utilization data for complex congenital heart defects (by age groups 18-24, 25-44, 45-64, 65+) and by type of care (G.P., Emergency Room and Out Patient visits, ICU and non-ICU hospitalization days) was multiplied by unit cost data [of $15.77, $309, $88, $3,001 and $1,005 respectively] (19) to provide treatment costs by age (by means of interpolation) for sCHD. In a similar fashion, utilization rates for Intermediate CHD (19) were used to estimate mCHD costs.

v) Next, mCHD costs were estimated for the 0-17 year old’s by multiplying the estimates for sCHD by 60.3%, representing the ratio of mCHD to sCHD costs in 18 year olds.

w) The resultant annual costs by one year age gradations were multiplied by the percentage surviving to that age (see section r) to give the estimated annual costs by age. Finally, this was discounted using a 3% annual rate to calculate the estimated lifetime costs of sCHD and mCHD by gender and time of diagnosis.

**Utilities from fetal or neonatal losses.**

x) Disability Weights (DW) of 0.08 were imputed from parental averages (20) to the mother to be for ten years for miscarriages (21), stillbirths (21) and neonatal deaths and for two years for abortions (20,23). All these DW were adjusted by the age specific health status of the mother-to-be.

**Utilities of parents to having a child with CHD**

y) The percentages of CHD births by neurological developmental disorder (NDD) severity (none, mild, moderate and severe) and time of diagnosis (prenatal and postnatal) was obtained from a recent cost-effectiveness study (20), where there was a greater prevalence of NDD in postnatally as opposed to prenatally diagnosed infants (16.7% vs 5.0%). Multiplication by the CHD birthrate in Israel of 1.147 per 1000 (8) provided an estimate of the numbers in each NDD category.

z) QALY losses of the mother were calculated by applying DWs (20) of 0.05, 0.12,0 .10, and 0.27 over the lifetime (adjusting for age specific non-NDD DW) for none, mild, moderate and severe NDD categories respectively.

aa) Disutility weights for surgery was assumed to be zero, as no parent would conceivably opt for a lower quality state related directly to not undergoing potentially life-saving surgery (16)

ab) We assigned a DW in comparison with parents of healthy children of 0.959 and 0.957 (24) for the QOL of fathers and mothers of CHD children. These were subsequently applied to the age and gender specific HSVs of the patients. We limited this application up to the time the child reached 18 years old.

**Utilities losses per CHD case.**

ac) Based on WHO Global Burden of Disease estimates (25) for persons with CHD who had NDD, DWs of 0.089, 0.144, and 0.220 were applied over the lifetime (adjusting for age specific non-CHD DW) for mild (mCHD), moderate (mCHD) and sCHD categories respectively.

ad) For persons without NDD, DWs (30) of 0.041, 0.072 and 0.251 were applied over the lifetime (adjusting for age specific non-CHD DW) for mild, moderate and severe levels of NDD respectively.

ae) The average DW of persons with sCHD and mCHD was calculated [from ab & ac] and applied to the age and gender specific background DW of the general population. In turn this was multiplied by the percentage of persons surviving every year as a result of prenatal, postnatal discovery of CHD in less than 24 hours and discovery after 24 hours.

af) Discounted lifetime HALE was calculated for all the gender and discovery of CHD timing categories. These were then subtracted from the discounted lifetime HALE of the average population in order to estimate the morbidity and mortality losses from CHD by discovery timing and gender.

ag) For each screening category (in addition to the null category) the numbers in each timing and gender category were multiplied by the relevant specific QALY losses in order to estimate the overall screening-specific QALY losses as a result of CHD.

**Intervention Costs were based as follows:**

ah) US and echocardiography intervention costs of $124.42 and $453 respectively were based on Ministry of Health national price data (26). DL costs, were based on a recent study (27) showing them to be 16.1% or $20.40 more (i.e. $144.82) than ultrasound in the diagnosis of colorectal cancer from colonoscopies.

ai) The cost of POX screening newborns, including provision for 13% repeated tests (28), amounted to $9.58, consisting of $0.21 oximeter & $0.08 probe costs in addition to $9.29 of labour costs (based on 9.8 minutes of nurses time (28) at Ministry of Health based on employment costs of a nurse with five years’ experience of $50.30 per hour.

aj) Miscarriage and Stillbirth costs of $6,172 and $5,273 were estimated from a UK meta-analysis (9). TOPs were based on Ministry costs (26) averaging 3,031 nis based on 67.3%, 31.0% and 1.7% (29) undergoing induced surgical abortion ($902), pharmaceutical abortion ($708) and late intra-amniotic injection ($1222) respectively. In addition, an estimated $5,273 of extra costs relating to counselling and work productivity losses were incurred (30). We assumed all TOP, whether voluntary or involuntary will be replaced by the mother to be having another child.

ak) We integrated into the final spreadsheet calculations the intervention costs, treatment costs and loss of QALYs for each of the CHD screening strategies in addition to a theoretical null scenario, where no screening is carried out.

**References for Appendix III (methodology):**

1. Khoshnood B, Lelong N, Houyel L, Bonnet D, Ballon M, Jouannic J-M, et al. Impact of prenatal diagnosis on survival of newborns with four congenital heart defects: a prospective, population-based cohort study in France (the EPICARD Study). BMJ Open 2017;7:e018285.

2. Eapen RS, Rowland DG, Franklin WH. Effect of prenatal diagnosis of critical left heart obstruction on perinatal morbidity and mortality. Am J Perinatol. 1998;15:237-42.

3. Fuchs IB, Müller H, Abdul-Khaliq H, Harder T, Dudenhausen JW, Henrich W. Immediate and long-term outcomes in children with prenatal diagnosis of selected isolated congenital heart

defects. Ultrasound Obstet Gynecol 2007;29: 38-43.

4. Slodki M, Rizzo G, Augustyniak A, Seligman NS, Zych-Krekora K, Respondek-Liberska M & The International Prenatal Cardiology Collaboration Group. Retrospective cohort study of prenatally and postnatally diagnosed coarctation of the aorta (CoA): prenatal diagnosis improves neonatal outcome in severe CoA, The Journal of Maternal-Fetal & Neonatal Medicine 2020; 33: 947-51.

5. Wolter A, Gebert M, Enzensberger C, Kawecki A, Stessig R, Degenhardt J et al. Outcome and Associated Findings in Individuals with Pre- and Postnatal Diagnosis of Tetralogy of Fallot (TOF) and Prediction of Early Postnatal Intervention. Ultraschall in Med 2018; doi <https://doi.org/10.1055/a-0753-0008> (Accessed 22nd January 2024).

6. Peake LK, Draper ES, Budd JLS, Field D. Outcomes when congenital heart disease is diagnosed antenatally versus postnatally in the UK: a retrospective population-based study. BMC Pediatrics 2015: 15:58.

7. Lim JSL, McCrindle BW, Smallhorn JF, Golding F, Caldarone CA, Taketazu M et al. Clinical Features, Management, and Outcome of Children With Fetal and Postnatal Diagnoses of Isomerism Syndromes. Circulation. 2005; 112: 2454-61.

8. Israel Ministry of Health. Congenital Malformations in Israel (2000-2014), Quality checks and trends over time. Ministry of health, Jerusalem.

9. Ghosh J, Papadopoulou A, Devall AJ, Jeffery HC, Beeson LE, Do V et al. Methods for managing miscarriage: a network meta-analysis. Cochrane Database Syst Rev. 2021 ;6 :CD012602. doi: 10.1002/14651858.CD012602.pub2.

10. Central Bureau of Statistics. Statistical Abstract of Israel 2016 no. 67, Jerusalem. [Statistical Abstract of Israel 2016 - No.67](https://www.cbs.gov.il/en/publications/Pages/2021/Statistical-Abstract-of-Israel-2021-No-72.aspx). <https://www.cbs.gov.il/en/publications/Pages/2016/Statistical-Abstract-of-Israel-2016-No-67.aspx> (Accessed 22nd January 2024).

11. Central Bureau of Statistics. Statistical Abstract of Israel 2022 no. 73, Jerusalem. Statistical Abstract of Israel 2022 - No.73. <https://www.cbs.gov.il/en/publications/Pages/2022/Statistical-Abstract-of-Israel-2022-No-73.aspx> (Accessed 22nd January 2024).

12. Central Bureau of Statistics. Statistical Abstract of Israel 2021 no. 72, Jerusalem. Statistical Abstract of Israel 2022 - No.73. <https://www.cbs.gov.il/en/publications/Pages/2021/Statistical-Abstract-of-Israel-2021-No-72.aspx> (Accessed 22nd January 2024).

13. . Central Bureau of Statistics. Applications for Pregnancy Termination in 2019, 29th December 2020, Jerusalem. [05_20_434b.pdf (cbs.gov.il)](https://www.cbs.gov.il/he/mediarelease/DocLib/2020/434/05_20_434b.pdf) https://www.cbs.gov.il/en/mediarelease/Pages/2020/Applications-for-Pregnancy-Termination-in-2019.aspx (Accessed 22nd January 2024). (In Hebrew)

14. Arnaout R, Curran L, Zhao Y, Levine JC, Chinn E, Moon-Grady AJ. An ensemble of neural networks provides expert-level prenatal detection of complex congenital heart disease. Nature Medicine 2021;27: 882–91.

15. Mukerji A, Shafey A, Jain A, Cohen E, Shah PS, Sander B,Shah V. Pulse oximetry screening for critical congenital heart defects in Ontario, Canada: a cost-effectiveness analysis. Canadian Journal of Public Health 2020; 111:804–811 Supplement 2.

16. Han B, Tang Y, Qu X, Deng C, Wang X, Li J. Comparison of the 1-year survival rate in infants with congenital heart disease diagnosed by prenatal and postnatal ultrasound: a retrospective study. Medicine 2021;100:4 (e23325).

17. Best KE, Rankin J . Long-Term Survival of Individuals Born with Congenital Heart Disease: A Systematic Review and Meta-Analysis. J Am Heart Assoc. 2016;5: e002846

18. Benderly M, Buber J, Kalter-Leibovici O, Blieden L, Dadashev A, Lorber A, et al. for the Israeli Adult Congenital Heart Disease Research Group. Health Service Utilization Patterns Among Adults With Congenital Heart Disease. : A Population-Based Study. J Am Heart Assoc. 2021;10:e018037.

19. Ginsberg G, Kark J, Einav S. Is out-of-hospital resuscitation worthwhile? Cost-Utility Analysis of Cardiac Resuscitation Services in Jerusalem. Resuscitation 2015; 86;54-61.

20. Bak GS, Shaffer BL, Madriago E, Allen A, Kelly B, Caughey AB, Pereira L. Detection of fetal cardiac anomalies: cost-effectiveness of increased number of cardiac views. Ultrasound Obstet Gynecol 2020; 55: 758–67

21. Gravensteen IK, Helgadottir LB, Jacobsen E-M, Sandset PM, Ekeberg Ø. Long-term impact of intrauterine fetal death on quality of life and depression: a case-control study. BMC Pregnancy Childbirth 2012; 12: 43.

22. Turton P, Evans C, Hughes P. Long-term psychosocial sequelae of stillbirth: phase II of a nested case-control study. Arch Womens Ment Health 2009; 12: 35–41.

23. Kuppermann M, Nease RF, Learman LA, Gates E, Blumberg B, Washington AE. Procedure-related miscarriages and Down syndrome-affected births: implications for prenatal testing based on women’s preferences. Obstet Gynecol 2000; 96: 511–6.

24. Lawoko S, Soares JJF. Quality of life among parents of children with congenital heart disease, parents of children with other diseases and parents of healthy children. Quality of Life Research 2003;12: 655–66.

25. Global Health Data Exchange GHDx. Global Burden of Disease Study 2019 (GBD 2019) Disability Weights. <https://ghdx.healthdata.org/record/ihme-data/gbd-2019-disability-weights>. Accessed (accessed 2nd May 2022)

26. Ministry of Health Price List for Ambulatory and Hospitalization Services. Effective date: 01/10/2022. Ministry of Health, Jerusalem. https:// [www.gov.il/BlobFolder/](http://www.gov.il/BlobFolder/) dynamiccollectorresultitem/spl-moh-price-list-030722/he/files_databases_moh-price-list_2022_moh-Price-List-010722.xlsx (Accessed 22^nd^ January 2024)

27. Areia M, Mori Y, Correale L, Repici A, Bretthauer M, Sharma P et al. Cost-effectiveness of artificial intelligence for screening colonoscopy: a modelling study. Lancet Digit Health 2022; 4: e436–44

28. Reeder MR, Kim J, Nance A, Krikov S , Feldkamp ML, Randall H et al. Evaluating Cost and Resource Use Associated with Pulse Oximetry Screening for Critical Congenital Heart Disease: Empiric Estimates and Sources of Variation Birth Defects Research (Part A) 2015:103:962–71.

29. Efrati I. Israel’s Abortion Rate Falls as Birth Control, Traditional Mores Rise. Haaretz. May 13th 2018. <https://www.haaretz.com/israel-news/2018-05-13/ty-article/.premium/israeli-abortion-rate-falls-as-birth-control-traditional-mores-rise/0000017f-ed4c-d4a6-af7f-ffce608f0000> (accessed 16th April 2023).

30. Quenby S, Gallos ID, Dhillon-Smith RK, Podesek M, Stephenson MD, Fisher J et al. Miscarriage matters: the epidemiological, physical, psychological, and economic costs of early pregnancy loss. Lancet 2021; 397: 1658–67.

| **Appendix IV**: Infants surviving post-intervention (a) by serious CHD diagnosis (prenatal or postnatal: studies 2000-23)   \|  \|  \| Live \| Died \| % live \| Weight \| Live \| Died \| % live \| \| --- \| --- \| --- \| --- \| --- \| --- \| --- \| --- \| --- \| \|  \| Ref \| Births \|  \| births \| (b) \| Births \|  \| births \| \|  \|  \| (n) \|  \| surviving \|  \| (n) \|  \| surviving \| \|  \|  \|  \|  \|  \|  \|  \|  \|  \| \| AORTIC VALVE STENOSIS \|  \| 1 \| 1 \| 0% \| 3.3% \| 1 \| 1 \| 0% \| \| UNIVENTRICULAR HEART \|  \| 32 \| 17 \| 46.9% \| 1.2% \| 7 \| 3 \| 57.1% \| \| TRUNCUS ARTERIOSIS \|  \| 19 \| 8 \| 57.9% \| 1.2% \| 94 \| 8 \| 91.5% \| \| HLHS \|  \| 433 \| 125 \| 71.1% \| 4.3% \| 539 \| 206 \| 61.8% \| \| PULMONARY ATRESIA \|  \| 59 \| 9 \| 84.7% \| 3.3% \| 45 \| 5 \| 88.9% \| \| AVSD \|  \| 80 \| 11 \| 86.0% \| 6.1% \| 135 \| 15 \| 89.0% \| \| TOF \|  \| 106 \| 8 \| 92.9% \| 9.1% \| 134 \| 8 \| 94.4% \| \| COARCTATION OF AORTA \|  \| 116 \| 8 \| 93.1% \| 11.2% \| 221 \| 20 \| 91.0% \| \| LHO \|  \| 15 \| 1 \| 93.3% \| 2.3% \| 47 \| 9 \| 80.9% \| \| TGA \|  \| 368 \| 14 \| 96.2% \| 8.2% \| 951 \| 76 \| 92.0% \| \| TAPVR \|  \| 0 \| 0 \| n.a. \| 1.2% \| 1 \| 1 \| 0% \| \|  \|  \|  \|  \|  \|  \|  \|  \|  \| \| TOTAL (c) \|  \| 1229 \| 202 \| **88.3%** \| 51.6% \| 2175 \| 351 \| **87.0%** \| \|  \|  \|  \|  \|  \|  \|  \|  \|  \| \| HLHS: Hypoplastic Left Heart Syndrome \| \|  \|  \|  \|  \|  \|  \|  \| \| AVSD: Atrioventricular Septal Defect \| \|  \|  \|  \|  \|  \|  \|  \| \| TOF : Tetralogy of Fallot \|  \|  \|  \|  \|  \|  \|  \|  \| \| LHO: Left Heart Obstruction, excluding HLHS and Coarctation of Aorta. \| \| \| \| \| \|  \|  \|  \| \| TGA: Transposition of the Great Arteries \| \| \|  \|  \|  \|  \|  \|  \| \| TAPVR : Total Anomalous Pulmonary Venous Return \| \| \| \|  \|  \|  \|  \|  \| \|  \|  \|  \|  \|  \|  \|  \|  \|  \| \| 1. post intervention, discharge or up to one year. Just one study was based on five year mortality 2. based on Israel main text reference (37) 3. Weighted survival rate of all sCHDs. \| \| \| \| \| \| \| \| \| \|  \| \|  \|  \|  \|  \|  \|  \|  \| \|  \|  \|  \|  \|  \|  \|  \|  \|  \| \|  \|  \|  \|  \|  \|  \|  \|  \|  \| \|  \|  \|  \|  \|  \|  \|  \|  \|  \| \|  \|  \|  \|  \|  \|  \|  \|  \|  \| \|  \| \|  \|  \|  \|  \|  \|  \|  \|  \| **Appendix V**: Sensitivity and Specificity of Ultrasound studies (2015-2023) to detect Serious CHD. \| \| \| \| \| \| \| \| \| --- \| --- \| --- \| --- \| --- \| --- \| --- \| --- \| \|  \|  \|  \|  \|  \|  \|  \|  \| \| Under Study Conditions \|  \| **True Pos** \| **False Pos** \| **False Neg** \| **True Neg** \| **Sensitivity** \| **Specificity** \| \|  \|  \|  \|  \|  \|  \|  \|  \| \|  \|  \|  \|  \|  \|  \|  \|  \| \| Colosi \| 2015 \| 0 \| 0 \| 3 \| 5921 \| 0.0% \| 100.00% \| \| Wiechec \| 2015 \| 29 \| 0 \| 2 \| 1053 \| 93.5% \| 100.00% \| \| D'Antonio \| 2016 \| 2 \| 0 \| 8 \| 2118 \| 20.0% \| 100.00% \| \| Takita \| 2016 \| 1 \| 0 \| 1 \| 2006 \| 50.0% \| 100.00% \| \| Tudorache \| 2016 \| 21 \| 13 \| 5 \| 2869 \| 80.8% \| 99.55% \| \| De Robertis \| 2017 \| 24 \| 6 \| 6 \| 5307 \| 80.0% \| 99.89% \| \| Vellamkondo \| 2017 \| 7 \| 0 \| 5 \| 428 \| 58.3% \| 100.00% \| \| Garcia-Fernandez \| 2018 \| 4 \| 0 \| 0 \| 655 \| 100.0% \| 100.00% \| \| Kenkhuis \| 2018 \| 3 \| 0 \| 6 \| 5005 \| 33.3% \| 100.00% \| \| Sainz \| 2018 \| 9 \| 0 \| 1 \| 401 \| 90.0% \| 100.00% \| \| Zheng \| 2018 \| 28 \| 1 \| 2 \| 1561 \| 93.3% \| 99.94% \| \| Chen \| 2019 \| 52 \| 0 \| 14 \| 10228 \| 78.8% \| 100.00% \| \| Elbrashy \| 2019 \| 68 \| 2 \| 12 \| 3158 \| 85.0% \| 99.94% \| \| Erenel \| 2019 \| 6 \| 1 \| 0 \| 664 \| 100.0% \| 99.85% \| \| Duta \| 2021 \| 29 \| 0 \| 6 \| 6877 \| 82.9% \| 100.00% \| \|  \|  \|  \|  \|  \|  \|  \|  \| \| Total \|  \| 283 \| 23 \| 71 \| 48251 \| 79.9% \| 99.95% \| \|  \|  \|  \|  \|  \|  \|  \|  \| \| Notes: All screenings were between 11weeks+0 days and 13 weeks+6days gestation \| \| \| \| \| \| \| \| \|  \|  \|  \|  \|  \|  \|  \|  \| \| Under routine conditions \| \|  \|  \|  \|  \|  \|  \| \|  \|  \| **True Pos** \| **False Pos** \| **False Neg** \| **True Neg** \| **Sensitivity** \| **Specificity** \| \| Andrew* \| 2015 \| 1 \| 0 \| 2 \| 4418 \| 33.3% \| 100.00% \| \| Syngelaki* \| 2019 \| 112 \| 0 \| 90 \| 100795 \| 55.4% \| 100.00% \| \| Vayna* \| 2018 \| 23 \| 0 \| 6 \| 6016 \| 79.3% \| 100.00% \| \|  \|  \|  \|  \|  \|  \|  \|  \| \| Total \|  \| 136 \| 0 \| 98 \| 111229 \| 58.1% \| 100.0% \| | | | | | | | | | | | | | | | |
| --- | --- | --- | --- | --- | --- | --- | --- | --- | --- | --- | --- | --- | --- | --- | --- | --- | --- | --- | --- | --- | --- | --- | --- | --- | --- | --- | --- | --- | --- | --- | --- | --- | --- | --- | --- | --- | --- | --- | --- | --- | --- | --- | --- | --- | --- | --- | --- | --- | --- | --- | --- | --- | --- | --- | --- | --- | --- | --- | --- | --- | --- | --- | --- | --- | --- | --- | --- | --- | --- | --- | --- | --- | --- | --- | --- | --- | --- | --- | --- | --- | --- | --- | --- | --- | --- | --- | --- | --- | --- | --- | --- | --- | --- | --- | --- | --- | --- | --- | --- | --- | --- | --- | --- | --- | --- | --- | --- | --- | --- | --- | --- | --- | --- | --- | --- | --- | --- | --- | --- | --- | --- | --- | --- | --- | --- | --- | --- | --- | --- | --- | --- | --- | --- | --- | --- | --- | --- | --- | --- | --- | --- | --- | --- | --- | --- | --- | --- | --- | --- | --- | --- | --- | --- | --- | --- | --- | --- | --- | --- | --- | --- | --- | --- | --- | --- | --- | --- | --- | --- | --- | --- | --- | --- | --- | --- | --- | --- | --- | --- | --- | --- | --- | --- | --- | --- | --- | --- | --- | --- | --- | --- | --- | --- | --- | --- | --- | --- | --- | --- | --- | --- | --- | --- | --- | --- | --- | --- | --- | --- | --- | --- | --- | --- | --- | --- | --- | --- | --- | --- | --- | --- | --- | --- | --- | --- | --- | --- | --- | --- | --- | --- | --- | --- | --- | --- | --- | --- | --- | --- | --- | --- | --- | --- | --- | --- | --- | --- | --- | --- | --- | --- | --- | --- | --- | --- | --- | --- | --- | --- | --- | --- | --- | --- | --- | --- | --- | --- | --- | --- | --- | --- | --- | --- | --- | --- | --- | --- | --- | --- | --- | --- | --- | --- | --- | --- | --- | --- | --- | --- | --- | --- | --- | --- | --- | --- | --- | --- | --- | --- | --- | --- | --- | --- | --- | --- | --- | --- | --- | --- | --- | --- | --- | --- | --- | --- | --- | --- | --- | --- | --- | --- | --- | --- | --- | --- | --- | --- | --- | --- | --- | --- | --- | --- | --- | --- | --- | --- | --- | --- | --- | --- | --- | --- | --- | --- | --- | --- | --- | --- | --- | --- | --- | --- | --- | --- | --- | --- | --- | --- | --- | --- | --- | --- | --- | --- | --- | --- | --- | --- | --- | --- | --- | --- | --- | --- | --- | --- | --- | --- | --- | --- | --- | --- | --- | --- | --- | --- | --- | --- | --- | --- | --- | --- | --- | --- | --- | --- | --- | --- | --- | --- | --- | --- | --- | --- | --- | --- | --- | --- | --- | --- | --- | --- | --- | --- | --- | --- | --- | --- | --- | --- | --- | --- | --- | --- | --- | --- | --- | --- | --- | --- | --- | --- | --- | --- | --- | --- | --- | --- | --- | --- | --- | --- | --- | --- | --- | --- | --- | --- | --- | --- | --- | --- | --- | --- | --- | --- | --- | --- | --- | --- | --- | --- | --- | --- | --- | --- | --- | --- | --- | --- | --- | --- | --- | --- | --- | --- | --- | --- | --- | --- | --- | --- | --- | --- | --- | --- | --- | --- | --- | --- | --- | --- | --- | --- | --- | --- | --- | --- | --- | --- | --- | --- | --- | --- | --- | --- | --- | --- | --- | --- | --- | --- | --- | --- | --- | --- | --- | --- | --- | --- | --- | --- | --- | --- | --- | --- | --- | --- | --- | --- | --- | --- | --- | --- | --- | --- | --- | --- | --- | --- | --- | --- | --- | --- | --- | --- | --- | --- | --- | --- | --- | --- | --- | --- | --- | --- | --- | --- |
|  | | | | | | | | |  |  |  |  |  |  |  |
|  | | | | | | | | |  |  |  |  |  |  |  |
|  | | | | | | | | |  |  |  |  |  |  |  |
|  | | | | | | | | |  |  |  |  |  |  |  |
|  | | | | | | | | |  |  |  |  |  |  |  |
|  | | | | | | | | |  |  |  |  |  |  |  |
|  | | | | | | | | |  |  |  |  |  |  |  |
|  | | | | | | | | |  |  |  |  |  |  |  |
|  | | | | | | | | |  |  |  |  |  |  |  |
|  | | | | | | | | |  |  |  |  |  |  |  |
|  | | | | | | | | |  |  |  |  |  |  |  |
| \| **Appendix VI**: Sensitivity and Specificity of Ultrasound studies (2015-2023) to detect Minor CHD. \| \| \| \| \| \| \| \| \| --- \| --- \| --- \| --- \| --- \| --- \| --- \| --- \| \|  \|  \|  \|  \|  \|  \|  \|  \| \| Under study conditions \|  \| **True Pos** \| **False Pos** \| **False Neg** \| **True Neg** \| **Sensitivity** \| **Specificity** \| \|  \|  \|  \|  \|  \|  \|  \|  \| \|  \|  \|  \|  \|  \|  \|  \|  \| \| Wiechec \| 2015 \| 2 \| 0 \| 2 \| 1080 \| 50.0% \| 100.00% \| \| Takita \| 2016 \| 1 \| 1 \| 9 \| 1997 \| 10.0% \| 99.95% \| \| Tudorache \| 2016 \| 7 \| 4 \| 8 \| 2889 \| 46.7% \| 99.86% \| \| De Robertis \| 2017 \| 3 \| 0 \| 2 \| 5338 \| 60.0% \| 100.00% \| \| Vellamkondo \| 2017 \| 0 \| 0 \| 11 \| 429 \| 0.0% \| 100.00% \| \| Kenkhuis \| 2018 \| 1 \| 1 \| 2 \| 5010 \| 33.3% \| 99.98% \| \| Sainz \| 2018 \| 1 \| 0 \| 1 \| 409 \| 50.0% \| 100.00% \| \| Chen \| 2019 \| 13 \| 1 \| 49 \| 10231 \| 21.0% \| 99.99% \| \| Elbrashy \| 2019 \| 11 \| 3 \| 5 \| 3221 \| 68.8% \| 99.91% \| \| Erenel \| 2019 \| 3 \| 1 \| 1 \| 666 \| 75.0% \| 99.85% \| \| Duta \| 2021 \| 1 \| 0 \| 3 \| 6908 \| 25.0% \| 100.00% \| \|  \|  \|  \|  \|  \|  \|  \|  \| \| Total \|  \| 43 \| 11 \| 93 \| 38178 \| 31.6% \| 99.971% \| \|  \|  \|  \|  \|  \|  \|  \|  \| \| Notes: All screenings were between 11weeks+0 days and 13 weeks+6days gestation \| \| \| \| \| \| \| \| \|  \|  \|  \|  \|  \|  \|  \|  \| \| Under routine conditions \| \|  \|  \|  \|  \|  \|  \| \|  \|  \| **True Pos** \| **False Pos** \| **False Neg** \| **True Neg** \| **Sensitivity** \| **Specificity** \| \| Vayna* \| 2018 \| 3 \| 0 \| 3 \| 6039 \| 50.0% \| 100.00% \| \| Syngelaki* \| 2019 \| 5 \| 0 \| 182 \| 100810 \| 2.7% \| 100.00% \| \|  \|  \|  \|  \|  \|  \|  \|  \| \| Total \|  \| 8 \| 0 \| 185 \| 106849 \| 4.1% \| 100.00% \| | | | | | | | | |  |  |  |  |  |  |  |
|  | | | | | | | | |  |  |  |  |  |  |  |
|  | | | | | | | | |  |  |  |  |  |  |  |
|  | | | | | | | | |  |  |  |  |  |  |  |
|  | | | | | | | | |  |  |  |  |  |  |  |
|  | | | | | | | | |  |  |  |  |  |  |  |
|  | | | | | | | | |  |  |  |  |  |  |  |
|  | | | | | | | | |  |  |  |  |  |  |  |
|  | | | | | | | | |  |  |  |  |  |  |  |
|  | | | | | | | | |  |  |  |  |  |  |  |
| **Appendix V**II: Pulse Oximetry at Birth for serious CHD (based on 95% cut off) 2000-2022 | | | | | | | |  |  |  |  |  |  |  |  |
| (Including only studies that did not exclude positive prenatal diagnoses) | | | | | | |  |  |  |  |  |  |  |  |  |
|  |  | **True Pos** | **False Pos** | **False Neg** | **True Neg** | **Sensitivity** | **Specificity** |  |  |  |  |  |  |  |  |
|  |  |  |  |  |  |  |  |  |  |  |  |  |  |  |  |
| Arlettaz | 2006 | 17 | 7 | 3 | 3255 | 85.0% | 99.8% |  |  |  |  |  |  |  |  |
| Bhola | 2014 | 4 | 11 | 0 | 18786 | 100.0% | 99.9% |  |  |  |  |  |  |  |  |
| de Wahl | 2005 | 59 | 1 | 7 | 133 | 89.4% | 99.3% |  |  |  |  |  |  |  |  |
| de Wahl Granelli | 2009 | 19 | 68 | 10 | 39724 | 65.5% | 99.8% |  |  |  |  |  |  |  |  |
| Ewer | 2011 | 18 | 177 | 6 | 19854 | 75.0% | 99.1% |  |  |  |  |  |  |  |  |
| Gomez-Rodriguez | 2015 | 2 | 12 | 0 | 1023 | 100.0% | 98.8% |  |  |  |  |  |  |  |  |
| Jones | 2016 | 2 | 21 | 0 | 10237 | 100.0% | 99.8% |  |  |  |  |  |  |  |  |
| Kawalec | 2006 | 7 | 13 | 1 | 27179 | 87.5% | 100.0% |  |  |  |  |  |  |  |  |
| Klausner | 2017 | 0 | 4 | 1 | 10315 | 0.0% | 100.0% |  |  |  |  |  |  |  |  |
| Kochilas | 2013 | 1 | 5 | 0 | 7543 | 100.0% | 99.9% |  |  |  |  |  |  |  |  |
| Koppel | 2003 | 3 | 1 | 2 | 11275 | 60.0% | 100.0% |  |  |  |  |  |  |  |  |
| Meberg | 2008 | 27 | 297 | 8 | 49676 | 77.1% | 99.4% |  |  |  |  |  |  |  |  |
| Oakley | 2015 | 7 | 7 | 1 | 6314 | 87.5% | 99.9% |  |  |  |  |  |  |  |  |
| Ozalaka | 2017 | 6 | 1 | 4 | 8197 | 60.0% | 100.0% |  |  |  |  |  |  |  |  |
| Richmond | 2002 | 10 | 54 | 9 | 5553 | 52.6% | 99.0% |  |  |  |  |  |  |  |  |
| Ruangritnamchai | 2007 | 3 | 0 | 0 | 1844 | 100.0% | 100.0% |  |  |  |  |  |  |  |  |
| Tautz | 2010 | 9 | 9 | 2 | 3344 | 81.8% | 99.7% |  |  |  |  |  |  |  |  |
| Turska-Kmiec | 2012 | 15 | 14 | 4 | 51665 | 78.9% | 100.0% |  |  |  |  |  |  |  |  |
| Van Nienerk | 2016 | 1 | 1 | 1 | 998 | 50.0% | 99.9% |  |  |  |  |  |  |  |  |
| Zuppa | 2014 | 75 | 226 | 9 | 151 | 89.3% | 40.1% |  |  |  |  |  |  |  |  |
| Badawi | 2019 | 1 | 27 | 9 | 78505 | 10.0% | 100.0% |  |  |  |  |  |  |  |  |
| Saxena | 2015 | 22 | 6026 | 4 | 12957 | 84.6% | 68.3% |  |  |  |  |  |  |  |  |
| Garg | 2013 | 7 | 42 | 48 | 72597 | 12.7% | 99.9% |  |  |  |  |  |  |  |  |
| Lightfoot | 2017 | 0 | 4 | 0 | 720 |  | 99.4% |  |  |  |  |  |  |  |  |
|  |  |  |  |  |  |  |  |  |  |  |  |  |  |  |  |
| Total |  | 315 | 7,028 | 129 | 441,845 | 70.95% | 98.43% |  |  |  |  |  |  |  |  |

| **Appendix VIII**: Effects and Costs by Interventions and CHD type. | | |
| --- | --- | --- |
|  |  |  |

|  |  | US |  | US | Deep | Deep |
| --- | --- | --- | --- | --- | --- | --- |
|  | Null | retro | POX | retro | Learning | Learning |
|  |  | studies |  | & POX |  | & POX |
|  |  |  |  |  |  |  |
| **Serious CHD (sCHD)** |  |  |  |  |  |  |
| Viable at 12 weeks | 905 | 905 | 905 | 905 | 905 | 905 |
| Abortions (a) | 14 | 173 | 14 | 173 | 2778 | 2778 |
| Miscarried | 87 | 71 | 87 | 71 | 60 | 60 |
| Stillborn | 2 | 2 | 2 | 2 | 2 | 2 |
| Live Births | 802 | 659 | 802 | 659 | 560 | 560 |
| Undiscovered | 802 | 346 | 233 | 102 | 49 | 16 |
| Prenatal Diagnosis | 0 | 313 | 0 | 313 | 511 | 511 |
| Postnatal Diagnosis | 0 | 0 | 569 | 244 | 0 | 33 |
| Diagnosed (% of live births) | 0% | 47% | 71% | 85% | 91% | 97% |
| Cost per diagnosis (nis) |  | 866,922 | 348,298 | 505,494 | 683,018 | 660,394 |
|  |  |  |  |  |  |  |
|  |  |  |  |  |  |  |
| **Minor CHD (mCHD)** |  |  |  |  |  |  |
| Viable at 12 weeks | 1561 | 1,561 | 1561 | 1,561 | 1561 | 1561 |
| Abortions (a) | 24 | 41 | 24 | 41 | 41 | 41 |
| Miscarried | 48 | 48 | 48 | 48 | 48 | 48 |
| Stillborn | 5 | 5 | 5 | 5 | 5 | 5 |
| Live Births | 1485 | 1,473 | 1485 | 1,473 | 1473 | 1473 |
| Undiscovered | 1485 | 1,165 | 1485 | 1,165 | 1165 | 1165 |
| Prenatal Diagnosis | 0 | 308 | 0 | 308 | 308 | 308 |
| Postnatal Diagnosis | 0 | 0 | 0 | 0 | 0 | 0 |
| Diagnosed (% of live births) |  | 21% |  | 21% | 21% | 21% |
| Cost per diagnosis (nis) |  | 879,171 |  | 913,207 |  |  |

| **Appendix IX**: Additional Cost (million USD at 2022 prices) of DL-US (& POX) vs US (& POX) | | | | | | | |  |  |  |
| --- | --- | --- | --- | --- | --- | --- | --- | --- | --- | --- |
| (based on US “routine reports”: Sensitivity 58.1%, Specificity 100%) | | | | | | | |  |  |  |
| A: Routine | DL-US Specificity | | |  |  |  |  | |  |  |
| DL-US Sensitivity | **90%** | | | **92%** | **94%** | **96%** | **98%** | | **99%** | **100%** |
| **80%** | 40.2 | | | 30.4 | 20.5 | 10.7 | 0.9 | | -4.2 | -8.9 |
| **84%** | 37.8 | | | 28.0 | 18.2 | 8.3 | -1.5 | | -6.5 | -11.3 |
| **88%** | 35.4 | | | 25.6 | 15.8 | 6.0 | -3.9 | | -8.9 | -13.7 |
| **92%** | 33.0 | | | 23.2 | 13.4 | 3.6 | -6.3 | | -11.0 | -16.1 |
| **96%** | 30.7 | | | 20.8 | 11.0 | 1.2 | -8.6 | | -13.4 | -18.5 |
| **99%** | 28.9 | | | 19.0 | 9.2 | -0.6 | -10.4 | | -15.2 | -20.2 |
|  |  | | |  |  |  |  | |  |  |
| Screening Costs : US ($124) DL ($143) |  | | |  |  |  |  | |  |  |
|  |  | | |  |  |  |  | |  |  |
|  |  | | |  |  |  |  | |  |  |
| B. Routine high Cost | DL-US Specificity | | |  |  |  |  | |  |  |
| DL-US Sensitivity | **90%** | | | **92%** | **94%** | **96%** | **98%** | | **99%** | **100%** |
| **80%** | 60.1 | | | 50.0 | 40.2 | 30.4 | 20.5 | | 16.5 | 11.2 |
| **84%** | 57.7 | | | 47.9 | 37.8 | 28.0 | 18.2 | | 14.0 | 8.7 |
| **88%** | 55.4 | | | 45.5 | 35.7 | 25.9 | 15.8 | | 11.5 | 6.2 |
| **92%** | 53.0 | | | 43.2 | 33.3 | 23.5 | 14.3 | | 9.0 | 4.0 |
| **96%** | 50.6 | | | 40.8 | 31.0 | 21.1 | 11.8 | | 6.5 | 1.6 |
| **99%** | 48.8 | | | 39.0 | 29.2 | 19.3 | 9.9 | | 4.7 | -0.3 |
|  |  | | |  |  |  |  | |  |  |
| Screening Costs : US ($124) DL-US ($248) |  | | |  |  |  |  | |  |  |
| (based on US “research studies”: Sensitivity 79.9%, Specificity 99.95%) |  | | |  |  |  |  | |  |  |
| C. Routine High Performance & Costs | DL-US Specificity | | |  |  |  |  | |  |  |
| DL-US Sensitivity | **90%** | | | **92%** | **94%** | **96%** | **98%** | | **99%** | **100%** |
| **80%** | 54.2 | | | 44.4 | 34.5 | 24.7 | 14.9 | | 9.8 | 5.1 |
| **84%** | 51.8 | | | 42.0 | 32.1 | 22.3 | 12.5 | | 7.4 | 2.7 |
| **88%** | 49.4 | | | 39.6 | 29.8 | 19.9 | 10.1 | | 5.1 | 0.3 |
| **92%** | 47.0 | | | 37.2 | 27.4 | 17.6 | 7.7 | | 2.7 | -2.1 |
| **96%** | 44.6 | | | 34.8 | 25.0 | 15.2 | 5.4 | | 0.3 | -4.5 |
| **99%** | 42.9 | | | 33.0 | 23.2 | 13.4 | 3.6 | | -1.8 | -6.3 |
| Screening Costs : US ($280) DL-US ($301) | |  |  |  |  |  |  |  |  |  |
|  | |  |  |  |  |  |  |  |  |  |
| DL-US (& POX) costs less than US (& POX) | |  |  |  |  |  |  |  |  |  |

| **Appendix X**: Additional QALYS using DL-US (& POX) vs US (& POX) |
| --- |
| (based on US “routine reports” data: sensitivity 58.1%, specificity 100%)   \| **A & B.** \| DL-US Specificity \|  \|  \|  \|  \|  \|  \| \| --- \| --- \| --- \| --- \| --- \| --- \| --- \| --- \| \| DL-US Sensitivity \| **90%** \| **92%** \| **94%** \| **96%** \| **98%** \| **99%** \| **100%** \| \| **80%** \| 4 \| 202 \| 400 \| 599 \| 797 \| 896 \| 995 \| \| **84%** \| 185 \| 344 \| 582 \| 781 \| 979 \| 1,078 \| 1,177 \| \| **88%** \| 367 \| 566 \| 764 \| 963 \| 1,161 \| 1,240 \| 1,359 \| \| **92%** \| 549 \| 748 \| 946 \| 1,145 \| 1,343 \| 1,442 \| 1,541 \| \| **96%** \| 731 \| 930 \| 1,128 \| 1,327 \| 1,525 \| 1,624 \| 1,723 \| \| **99%** \| 868 \| 1,066 \| 1,265 \| 1,463 \| 1,661 \| 1,761 \| 1,860 \| |

(based on US research study data: sensitivity 79.9%, specificity 99.95%)

| **C.** | DL-US Specificity |  |  |  |  |  |  |
| --- | --- | --- | --- | --- | --- | --- | --- |
| DL-US Sensitivity | **90%** | **92%** | **94%** | **96%** | **98%** | **99%** | **100%** |
| **80%** | -984 | -786 | -587 | -389 | -190 | -91 | 8 |
| **84%** | -802 | -604 | -405 | -207 | -9 | 91 | 190 |
| **88%** | -620 | -422 | -223 | -25 | 173 | 273 | 372 |
| **92%** | -438 | -240 | -41 | 157 | 355 | 455 | 554 |
| **96%** | -256 | -58 | 141 | 339 | 537 | 637 | 736 |
| **99%** | -120 | 79 | 277 | 475 | 674 | 773 | 872 |

| DL-US (&POX) provide fewer QALYS than US (& POX) |  |
| --- | --- |

| **Appendix XI**: Meta Analysis of Miscarriage Rates (IUD) for Serious (ie: Critical or Major) CHD. | | | | | | | |  |
| --- | --- | --- | --- | --- | --- | --- | --- | --- |
|  |  |  |  |  |  |  |  |  |
|  |  |  | Time of | Fetal |  | Viable | Mis- | Mis- |
|  | Ref | Ref | Diagnosis | CHD | Aborted | Fetus | Carriage | Carriage |
|  |  | year | weeks | (n) | (n) | (n) | (n) | (%) |
|  |  |  |  |  |  |  |  |  |
| Vinals |  | 2008 | 12.5 | 3 | 1 | 2 | 2 | 100.0% |
| Tudorache |  | 2016 | 13.5 | 27 | 20 | 7 | 4 | 57.1% |
| Michailiadis |  | 2001 | 17.2 | 6 | 4 | 2 | 1 | 50.0% |
| Eleftheriades (a) |  | 2012 | 12.5 | 20 | 15 | 5 | 2 | 40.0% |
| Chen |  | 2008 | 17.5 | 8 | 5 | 3 | 1 | 33.3% |
| Grande |  | 2012 | 14.4 | 38 | 27 | 11 | 3 | 27.3% |
| Todros |  | 1997 | 26.4 | 10 | 2 | 8 | 1 | 12.5% |
| Bull |  | 1999 | 20.0 | 738 | 433 | 305 | 38 | 12.5% |
| Waern |  | 2021 | 20.0 | 112 | 59 | 53 | 6 | 11.3% |
| Ozkutlu |  | 2005 | 27.6 | 37 | 14 | 23 | 2 | 8.7% |
| Syngelaki |  | 2011 | 22.1 | 79 | 37 | 42 | 3 | 7.1% |
| Jin |  | 2021 | 24.5 | 124 | 94 | 30 | 2 | 6.7% |
| Xie |  | 2017 | 21.0 | 59 | 41 | 18 | 1 | 5.6% |
| Qiu |  | 2020 | 24.5 | 683 | 493 | 190 | 9 | 4.7% |
| Jorgensen |  | 2014 | 20.0 | 84 | 51 | 33 | 1 | 3.0% |
| Gabriel |  | 2002 | 14.2 | 35 | 31 | 4 | 0 | 0.0% |
| Luck |  | 1992 | 19.7 | 24 | 6 | 18 | 0 | 0.0% |
| Vanya |  | 2018 | 14.2 | 23 | 18 | 5 | 0 | 0.0% |
| Orlandi |  | 2014 | 13.5 | 19 | 13 | 6 | 0 | 0.0% |
| Weiner |  | 2008 | 13.6 | 14 | 12 | 2 | 0 | 0.0% |
| Rustico |  | 2000 | 14.3 | 11 | 10 | 1 | 0 | 0.0% |
| Kenkuis |  | 2018 | 17.0 | 9 | 4 | 5 | 0 | 0.0% |
| Erenel |  | 2019 | 12.9 | 7 | 3 | 4 | 0 | 0.0% |
| Colosi |  | 2015 | 22.0 | 3 | 0 | 3 | 0 | 0.0% |
| McAuliff |  | 2005 | 15.0 | 2 | 0 | 2 | 0 | 0.0% |
| Vellamkondu |  | 2017 | 15.8 | 2 | 1 | 1 | 0 | 0.0% |
|  |  |  |  |  |  |  |  |  |
| TOTAL |  |  |  | 2,177 | 1,394 | 783 | 76 | **9.7%** |
|  |  |  |  |  |  |  |  |  |
| (a) includes two embro-reductions of twin fetuses | | | |  |  |  |  |  |
| Note: Miscarriage Rate excluding the four largest studies is 9.80% | | | | | |  |  |  |

| **Appendix XII**: Meta Analysis of Miscarriage Rates (IUD) for Minor CHD. | | | | | |  |  |  |
| --- | --- | --- | --- | --- | --- | --- | --- | --- |
|  |  |  |  |  |  |  |  |  |
|  |  |  | Time of | Fetal |  | Viable |  | Miscarriage |
|  | Ref | Ref | Diagnosis | CHD | Aborted | Fetus | Miscarriages | Rate |
|  |  | (year} | ( weeks) | (n) | (n) | (n) | (n) | (%) |
|  |  |  |  |  |  |  |  |  |
| Zalel |  | 2016 | 12.7 | 12 | 11 | 1 | 1 | 100.0% |
| Vellamkondu |  | 2017 | 12.5 | 3 | 0 | 3 | 1 | 33.3% |
| Luck |  | 1992 | 19.0 | 5 | 0 | 5 | 1 | 20.0% |
| Waern |  | 2021 | 19.4 | 7 | 0 | 7 | 1 | 14.3% |
| Orlandi |  | 2014 | 15.5 | 11 | 0 | 11 | 1 | 9.1% |
| Qiu |  | 2020 | 24.5 | 452 | 290 | 162 | 13 | 8.0% |
| Syngelaki |  | 2011 | 23.0 | 28 | 2 | 26 | 2 | 7.7% |
| Xie |  | 2017 | 21.0 | 51 | 26 | 25 | 1 | 4.0% |
| Jin |  | 2021 | 24.5 | 646 | 107 | 539 | 5 | 0.9% |
| Tudorache |  | 2016 | 12.2 | 12 | 3 | 9 | 0 | 0.0% |
| Vanya |  | 2018 | 16.3 | 8 | 2 | 6 | 0 | 0.0% |
| Grande |  | 2012 | 19.0 | 7 | 5 | 2 | 0 | 0.0% |
| Todros |  | 1997 | 26.8 | 7 | 0 | 7 | 0 | 0.0% |
| Ozkutlu |  | 2005 | 27.6 | 5 | 0 | 5 | 0 | 0.0% |
| Chen |  | 2008 | 15.9 | 4 | 2 | 2 | 0 | 0.0% |
| Weiner |  | 2008 | 19.9 | 4 | 2 | 2 | 0 | 0.0% |
| Gabriel |  | 2002 | 15.3 | 3 | 2 | 1 | 0 | 0.0% |
| Kenkuis |  | 2018 | 16.5 | 3 | 0 | 3 | 0 | 0.0% |
| Bull |  | 1999 | 20.0 | 2 | 0 | 2 | 0 | 0.0% |
| Eleftheriades |  | 2012 | 12.5 | 6 | 1 | 5 | 0 | 0.0% |
| Erenel |  | 2019 | 12.3 | 2 | 0 | 2 | 0 | 0.0% |
| Rustico |  | 2000 | 14.3 | 2 | 0 | 2 | 0 | 0.0% |
|  |  |  |  |  |  |  |  |  |
| TOTAL |  |  | 23.7 | 1,280 | 453 | 827 | 26 | **3.14%** |

**Appendix XIII**: Sensitivity and Specificity of Ultrasound studies (2005-2014) to detect Serious CHD.

Under study conditions:

|  | Ref | Year | True Pos | False Pos | False Neg | True Neg | Sensitivity | Specificity |
| --- | --- | --- | --- | --- | --- | --- | --- | --- |

| McAuliffe | |  | 2005 | 0 | 0 | 1 | 273 | 0.0% | 100.0% |
| --- | --- | --- | --- | --- | --- | --- | --- | --- | --- |
| Cedergren | |  | 2006 | 0 | 0 | 2 | 2,706 | 0.0% | 100.0% |
| Srisupundit | |  | 2006 | 2 | 0 | 0 | 595 | 100.0% | 100.0% |
| Vimpelli | |  | 2006 | 1 | 0 | 1 | 582 | 50.0% | 100.0% |
| Dane | |  | 2007 | 0 | 0 | 1 | 1,289 | 0.0% | 100.0% |
| Lombardi | |  | 2007 | 0 | 0 | 3 | 605 | 0.0% | 100.0% |
| Li | |  | 2007 | 1 | 0 | 1 | 2,226 | 50.0% | 100.0% |
| Vinals | |  | 2008 | 3 | 0 | 2 | 30 | 60.0% | 100.0% |
| Chen (control) | |  | 2008 | 0 | 0 | 10 | 3,683 | 0.0% | 100.0% |
| Chen (study) | |  | 2008 | 5 | 5 | 0 | 3,939 | 100.0% | 99.9% |
| Oztekin | |  | 2009 | 0 | 0 | 2 | 1,028 | 0.0% | 100.0% |
| Benasar * | |  | 2009 | 7 | 0 | 0 | 52 | 100.0% | 100.0% |
| Sinkovskya | |  | 2010 | 4 | 0 | 1 | 95 | 80.0% | 100.0% |
| Krapp | |  | 2011 | 17 | 0 | 2 | 671 | 89.5% | 100.0% |
| Volpe | |  | 2011 | 19 | 5 | 6 | 4,415 | 76.0% | 99.89% |
| Jacobsen | |  | 2011 | 3 | 0 | 24 | 9,297 | 11.1% | 100.0% |
| Syngelaki | |  | 2011 | 28 | 0 | 62 | 44,769 | 31.1% | 100.0% |
| Becker | |  | 2012 | 7 | 0 | 8 | 6,529 | 46.7% | 100.0% |
| Novotna | |  | 2012 | 1 | 0 | 11 | 8,877 | 8.3% | 100.0% |
| Grande | |  | 2012 | 25 | 0 | 20 | 13,678 | 55.6% | 100.0% |
| Eleftheriadis | |  | 2012 | 11 | 0 | 1 | 3,743 | 91.7% | 100.0% |
| Wang | |  | 2013 | 4 | 0 | 1 | 2,817 | 80.0% | 100.0% |
| Orlandi | |  | 2014 | 16 | 0 | 4 | 4,010 | 80.0% | 100.0% |
|  |  | |  |  |  |  |  |  |  |
| Total |  | |  | 154 | 10 | 163 | 115,909 | 48.6% | 99.99% |
|  |  | |  |  |  |  |  |  |  |
| * adjusted by omitting a few observations over 15 weeks gestation | | | | | | | |  |  |
| n.a. Not available since 108 false positive results could not be classified as serious or minor CHD. | | | | | | | | | |
| Notes: All screenings were between 12weeks+0 days and 13 weeks+3 days gestation | | | | | | | | |  |
|  |  | |  |  |  |  |  |  |  |
| Routine conditions | | |  |  |  |  |  |  |  |
|  | Ref | | Year | True Pos | False Pos | False Neg | True Neg | Sensitivity | Specificity |
| Abu-Rustum |  | | 2010 | 5 | 1 | 1 | 1,355 | 83.3% | 99.9% |
| Hartge |  | | 2011 | 66 | 0 | 10 | 3,145 | 86.8% | 100.0% |
|  |  | |  |  |  |  |  |  |  |
| Total |  | |  | 71 | 1 | 11 | 4,500 | 86.6% | 99.98% |
|  |  | |  |  |  |  |  |  |  |

**Appendix XIV**: Sensitivity and Specificity of Ultrasound studies (2005-2014) to detect Minor CHD.

| Under study conditions | |  |  |  |  |  |  |  |
| --- | --- | --- | --- | --- | --- | --- | --- | --- |
|  | Ref | Year | True Pos | False Pos | False Neg | True Neg | Sensitivity | Specificity |

| Cedergren |  | 2006 | 0 | 0 | 14 | 2694 | 0.0% | 100.0% |
| --- | --- | --- | --- | --- | --- | --- | --- | --- |
| Vimpelli |  | 2006 | 0 | 0 | 4 | 580 | 0.0% | 100.0% |
| Dane |  | 2007 | 1 | 0 | 2 | 1287 | 33.3% | 100.0% |
| Li |  | 2007 | 0 | 0 | 3 | 2225 | 0.0% | 100.0% |
| Vinals |  | 2008 | 1 | 3 | 0 | 31 | 100.0% | 91.2% |
| Chen (control) |  | 2008 | 2 | 0 | 5 | 3686 | 28.6% | 100.0% |
| Chen (study) |  | 2008 | 2 | 0 | 5 | 3942 | 28.6% | 100.0% |
| Oztekin |  | 2009 | 0 | 0 | 1 | 1029 | 0.0% | 100.0% |
| Benasar * |  | 2009 | 3 | 2 | 1 | 53 | 75.0% | 96.4% |
| Sinkovskya |  | 2010 | 1 | 0 | 1 | 98 | 50.0% | 100.0% |
| Volpe |  | 2011 | 7 | 1 | 6 | 4431 | 53.8% | 100.0% |
| Jacobsen |  | 2011 | 9 | 0 | 16 | 9299 | 36.0% | 100.0% |
| Syngelaki |  | 2011 | 1 | 0 | 17 | 44841 | 5.6% | 100.0% |
| Novotna |  | 2012 | 0 | 0 | 3 | 8886 | 0.0% | 100.0% |
| Grande |  | 2012 | 3 | 0 | 77 | 13643 | 3.8% | 100.0% |
| Eleftheriadis |  | 2012 | 2 | 0 | 4 | 3749 | 33.3% | 100.0% |
| Wang |  | 2013 | 0 | 0 | 2 | 2820 | 0.0% | 100.0% |
| Orlandi |  | 2014 | 5 | 0 | 7 | 4018 | 41.7% | 100.0% |
|  |  |  |  |  |  |  |  |  |
| Total |  |  | 37 | 6 | 168 | 107,312 | 18.0% | 99.994% |
|  |  |  |  |  |  |  |  |  |
| * adjusted by omitting a few observations over 15 weeks gestation | | | | | | |  |  |
| Note: All screenings were between 12weeks+0 days and 13 weeks+3 days gestation | | | | | | | |  |
|  |  |  |  |  |  |  |  |  |
| Routine conditions | |  |  |  |  |  |  |  |
|  | Ref | Year | True Pos | False Pos | False Neg | True Neg | Sensitivity | Specificity |
| Abu-Rustum |  | 2010 | 3 | 0 | 2 | 1357 | 60.0% | 100.0% |
| Hartge |  | 2011 | 3 | 0 | 3 | 3215 | 50.0% | 100.0% |
|  |  |  |  |  |  |  |  |  |
|  |  |  | 6 | 0 | 5 | 4572 | 54.5% | 100.0% |

| **Appendix XV**: Survival Rates by CHD Diagnosis. | | | |  |  |  |  |  |
| --- | --- | --- | --- | --- | --- | --- | --- | --- |
|  |  |  |  |  |  |  |  |  |
| MINOR CHD |  | 1yr |  | 5yr |  | 10yr |  | 15yr |
|  |  |  |  |  |  |  |  |  |
| Atrial Septal Defect |  | 90.9% |  | 90.6% |  | 90.0% |  | 89.7% |
| Pulmonary Valve Stenosis |  | 94.2% |  | 93.6% |  | 93.6% |  | 93.6% |
| VSD |  | 94.3% |  | 94.2% |  | 92.4% |  | 92.4% |
| **TOTAL (a)** |  | **92.2%** |  | **91.9%** |  | **91.0%** |  | **90.8%** |
|  |  |  |  |  |  |  |  |  |
| SEVERE CHD |  | 1yr |  | 5yr |  | 10yr |  | 15yr |
|  |  |  |  |  |  |  |  |  |
| Aortic Valve atresia/stenosis |  | 83.3% |  | 81.7% |  | 81.7% |  | 80.0% |
| AVSD |  | 77.4% |  | 71.9% |  | 71.7% |  | 70.8% |
| Coartication of Aorta |  | 82.7% |  | 80.2% |  | 79.7% |  | 79.6% |
| Common Arterial Trunk |  | 69.8% |  | 65.9% |  | 59.1% |  | 59.1% |
| Ebstein’s Anomaly |  | 72.9% |  | 66.0% |  | 66.0% |  | 64.1% |
| HLHS |  | 48.0% |  | 43.9% |  | 43.9% |  | 39.9% |
| Pulmonary Valve Atresia |  | 50.0% |  | 43.7% |  | 41.8% |  | 40.1% |
| Single Ventricle |  | 66.4% |  | 57.7% |  | 57.7% |  | 50.2% |
| Tetralogy of Fallot |  | 86.8% |  | 86.7% |  | 85.2% |  | 85.1% |
| TAPR |  | 67.2% |  | 60.6% |  | 60.6% |  | 60.6% |
| TGV |  | 81.8% |  | 79.1% |  | 76.0% |  | 75.3% |
| Tricuspid Atresia |  | 68.8% |  | 63.3% |  | 60.7% |  | 58.4% |
| **TOTAL (b)** |  | **57.3%** |  | **54.9%** |  | **54.0%** |  | **52.8%** |
|  |  |  |  |  |  |  |  |  |
| AVSD: Atrioventricular Septal Defect | | |  |  |  |  |  |  |
| HLHS: Hypoplastic Left Heart Syndrome |  |  |  |  |  |  |  |  |
| TAPR: Total Anomalous Pulmonary Return | | | |  |  |  |  |  |
| TGV: Transposition of the Great Vessels | | | |  |  |  |  |  |
| Based on pooled estimates from a meta-analysis (main references 59). | | | | |  |  |  |  |
| (a) weighted by Israeli prevalence data (main references 71) | | | |  |  |  |  |  |
| (b) weighted by Israel national prevalence data (main references 65) | | | | |  |  |  |  |
